# Biologically informative not available deconvolution excavates hidden features in proteomics datasets

**DOI:** 10.1093/bib/bbag582

**Published:** 2026-10-26

**Authors:** Weiheng Guo, Wenyi Jin, Jieyi Zheng, Jian Zhang, Xikang Feng, Lingxi Chen, Liang Zhang

**Affiliations:** Department of Biomedical Sciences, College of Biomedicine, City University of Hong Kong, Tat Chee Ave, Kowloon, Hong Kong SAR 999077, China; Department of Biomedical Sciences, College of Biomedicine, City University of Hong Kong, Tat Chee Ave, Kowloon, Hong Kong SAR 999077, China; Department of Orthopaedics, Renmin Hospital of Wuhan University, Wuhan University, Zhangzhidong Road, Wuhan 430060, China; School of Software, Northwestern Polytechnical University, West Youyi Road, Xi’an 710072, China; Department of Pharmaceutical and Artificial-Intelligence Sciences, School of Medicine, Shanghai Jiao Tong University, South Chongqing Road, Shanghai 200025, China; School of Software, Northwestern Polytechnical University, West Youyi Road, Xi’an 710072, China; Department of Biomedical Sciences, College of Biomedicine, City University of Hong Kong, Tat Chee Ave, Kowloon, Hong Kong SAR 999077, China; Department of Biomedical Sciences, College of Biomedicine, City University of Hong Kong, Tat Chee Ave, Kowloon, Hong Kong SAR 999077, China

**Keywords:** clinical proteomics, missing value analysis, biomarker discovery, protein–protein interaction networks

## Abstract

Missing values [not available (NAs)] in proteomics arise from true absence/low abundance and technical factors. Conventional imputation often obscures disease signals by ignoring the biological information inherent in protein absence. We present biologically informative NA deconvolution (BIND), an adaptive neighborhood-based workflow that classifies NAs as biological or technical. BIND binarizes matrices to encode presence/absence and uses context-aware *k*-nearest neighbors with Gaussian kernels to deconvolve NA origins, enabling clustering, biomarker discovery, and an NA-weighted correlation for protein–protein interaction (PPI) inference. In a simulation derived from the CCLE949 dataset, BIND achieved an Area Under the Curve (AUC) of 0.93, with *k* = 10 and *γ* = 3 selected for subsequent Cancer Cell Line Encyclopedia (CCLE) analyses, and remained robust across biological-to-technical NA ratios ranging from 3:1 to 1:3. In hepatocellular carcinoma tissues (*n* = 248), BIND distinguished tumor from adjacent normal, identified candidate biomarkers, and detected cancer-specific PPIs enriched in spliceosome and DNA replication pathways. A public BIND web server offers interactive visualization and supports single-cell proteomics, enhancing accessibility. Overall, BIND transforms NA patterns from a liability into biologically informative features for clustering, endotype discovery, biomarker identification, and PPI analysis.

## Introduction

Proteomics underpins modern research fields, including biology, medicine, and environmental science [1]. A prominent challenge in clinical proteomics includes the pervasive missing values introduced by sample processing limitations and instrument detection constraints [2]. These missing values are often presented as “NA” (not available), which complicates the subsequent extraction of biological insights from the datasets. For example, NAs degrade matrix completeness and impede statistical analyses, such as principal component analysis and hierarchical clustering [3, 4]. Despite deeper coverage from improved mass spectrometry platforms, the NA burden typically increases as the cohort size grows [5].

Missing values in proteomics are often categorized as missing completely at random (MCAR), missing at random (MAR), and missing not at random (MNAR) [6]. In practice, MCAR/MAR tend to be technical, whereas MNAR often reflects biological scarcity and left-censoring near detection thresholds [7–9]. However, it remains challenging to deconvolve the NA natures, which are crucial for trustworthy analysis and decision-making in modern proteomics.

To address the challenges brought by missing values, data imputation is a common strategy [10–12]. This involves using statistical methods to estimate and impute missing values, thereby enabling subsequent data analysis. Imputation methods can be classified into naive imputation, feature-based imputation, and global-based imputation [9]. Deep learning model-based imputation methods have also been developed recently [13]. Despite producing a complete data matrix, imputation does not address the underlying cause of missingness and overlooks that true absence/low abundance can carry diagnostic value to distinguish disease activity [14], including cancer progression [15]. Imputation can obscure the diagnostic value of such “hallmark” absence, which is often underappreciated and lacks comprehensive investigation. Notably, binarized analyses have been adopted in single-cell transcriptomics to help the cell clustering analysis [16]. However, the report failed to systematically elucidate the biological significance of the missing values. We reasoned that NA patterns in proteomics could be more informative, as proteins are the actual executors of biological activities.

We therefore examined multiple proteomics datasets and observed that NA distributions alone segregate tissues of origin. To systematize this, we developed biologically informative NA deconvolution (BIND), which deconvolves NAs as biological or technical using adaptive neighborhood modeling and exposes their downstream utility through interactive visualization. In large-scale and clinical contexts, BIND revealed protein “absence hallmarks” in cancer tissues, aided biomarker discovery, and strengthened protein complex inference. Altogether, BIND offers a principled, interpretable approach for leveraging NA structure to extract biological insights.

## Materials and methods

### Problem formulation

Missing values (NAs) in proteomics are commonly viewed as noise to be removed via imputation. This approach is fundamentally limited, as it ignores the distinct biological meaning behind NAs: a value can be missing because the protein is biologically absent or due to a technical detection failure. Imputing these two types with the same mathematical approach creates analytical bias: filling a true biological absence with an estimated positive value introduces false signals, while treating a technical miss as a zero hides true expression.

We therefore reframe the core problem: treat NAs as valuable biological information rather than a burden in data analysis. To solve this, we introduce BIND, a framework that treats NAs as primary data. We first reveal the biological significance of NAs through their statistical analysis and visualization (Fig. 1A). Next, BIND aims to (i) classify NAs as biological or technical (Fig. 1B) and (ii) apply NA classification in downstream sample grouping, biomarker identification, and protein–protein interaction (PPI) analysis (Fig. 1C). This shifts the paradigm from filling gaps to decoding the information within them.

**Figure 1:**
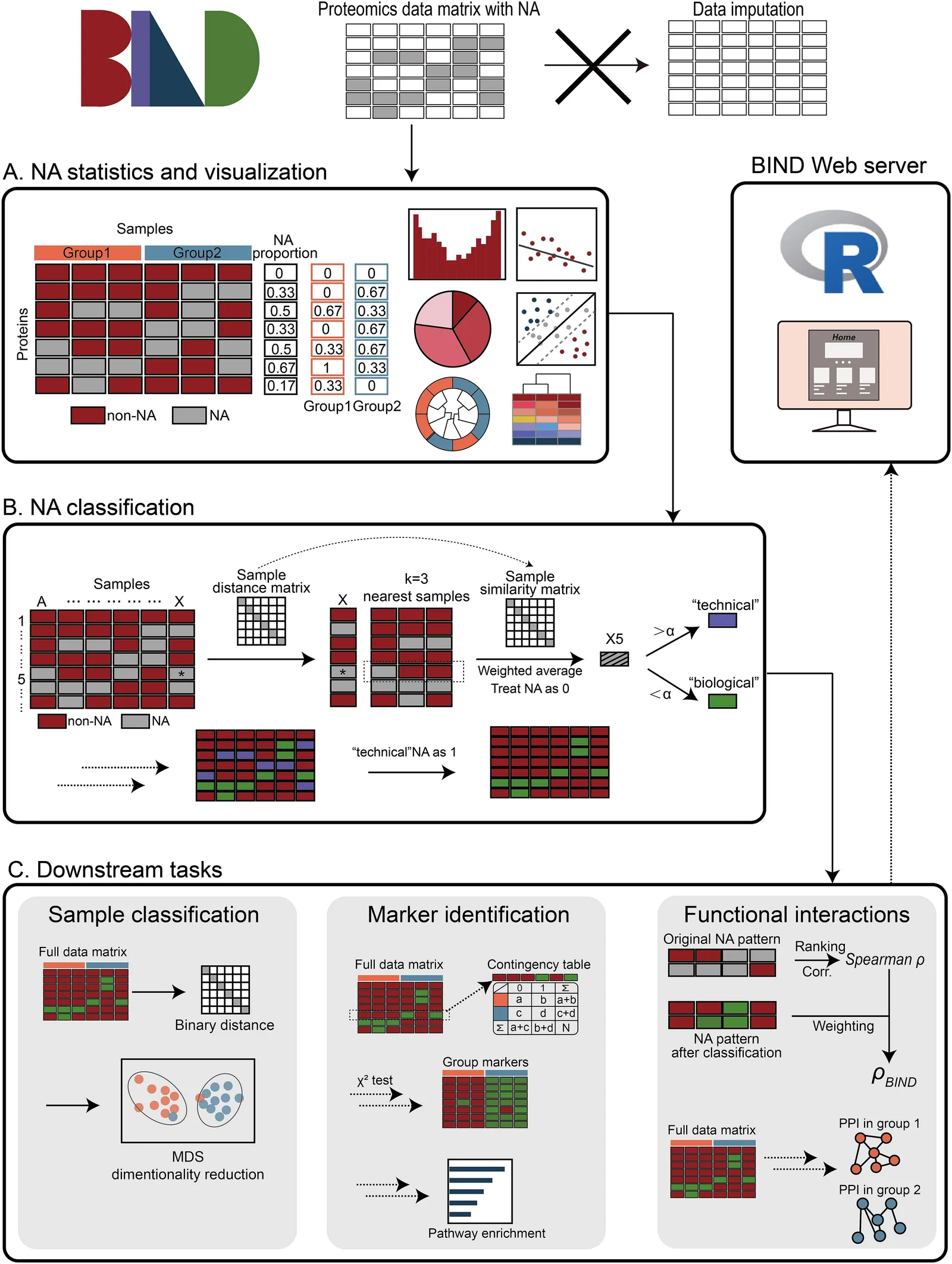
Method overview. (A) NA statistics and visualization. Data were converted into binary format, and the proportion of missing proteins was calculated. Statistical analysis and visualization were performed using methods including heatmaps, pie charts, and scatter plots. (B) Deconvolution of NA nature. Missing values were classified as “biological” or “technical” based on the features of *k*NN. “Technical” NA was restored to 1 (indicating the protein is present) because a value should have been present at this position but was not detected. (C) Downstream tasks. The obtained data can be utilized for multiple downstream biological tasks. Examples include sample dimensionality reduction and clustering based on binary data distances, identification of group-specific biomarkers, and Spearman’s correlation analysis with missing value assistance.

The BIND core algorithm requires only a protein expression matrix containing numerical expression values and NAs, where columns represent samples and rows represent proteins. BIND labels each NA position in the result matrix with a category (“biological” or “technical”). For downstream analyses involving sample clustering and biomarker identification, grouped sample data (e.g. cancer vs. normal) need to be provided.

### NA statistics and visualization

To uncover the intrinsic meaning of NAs, we first conduct a statistical analysis and visualization of NAs in the data to explore their potential biological significance. Given a protein expression matrix * X *, where $ {x}_{i,u} $ is the expression of protein * u * in sample * i *, we define the proportion of NA for a protein *u* as


(1) \begin{equation*} p\left(x=\phi \right)=\frac{\sum_{i=1}^n\delta \left({x}_{i,u}, \phi \right)}{n}, \end{equation*}

where we use *ϕ* to denote an NA in the protein matrix, and *n* is the sample number. *δ* is the Kronecker delta function and is defined as


(2) \begin{equation*} \delta \left(a,b\right)=\left\{\begin{array}{@{}c}1\kern0.75em a=b\\{}0\kern0.75em a\ne b\end{array}\right. \end{equation*}

An NA proportion of 0 $p\left(x=\phi \right)=0$ indicates that the protein is present in all samples, while an NA proportion of 1 $p\left(x=\phi \right)=1$ means it is absent in all samples. This method enables the calculation of NA proportions across the entire dataset or within specific sample groups, allowing for a detailed analysis of missing data patterns for each protein.

We binarize the dataset by replacing NA with 0 and other values with 1, allowing for the treatment of the data as asymmetric binary data [17], i.e. transfer ${x}_{i,u}$ to ${\overline{x}}_{i,u}\in \left\{0,1\right\}$. The original data matrix *X* is transformed into a binary data matrix $\overline{X}$. This enables the calculation of the Jaccard distance between samples, facilitating the analysis of similarity and dissimilarity in the presence of NAs [18]


(3) \begin{equation*} d{\left(i,j,\overline{X}\right)}_{\mathrm{Jaccard}}=\frac{m-{\sum}_{u=1}^m\delta \left({\overline{x}}_{i,u},{\overline{x}}_{j,u}\right)}{m-{\sum}_{u=1}^m\delta \left({\overline{x}}_{i,u},0\right)\bullet \delta \left({\overline{x}}_{j,u},0\right)\kern0.75em }, \end{equation*}

where *i* and *j* are two samples, *u* is the protein, and *m* is the total number of proteins. These calculated distances are then used for hierarchical clustering of samples, to show that NA pattern can discern different types of samples.

### Core algorithm of BIND

#### NA classification

To use the available observed data without prior imputation, we calculated the distance between each pair of samples using proteins with nonmissing expression values in both samples. Specifically, the distance between samples *i* and *j* in the protein expression matrix *X* is calculated as


(4) \begin{equation*} d{\left(i,j,X\right)}_{\mathrm{Euclidean}}=\sqrt{\frac{m}{\mid{O}_{ij}\mid}\sum_{u\epsilon{O}_{ij}}{\left({x}_{i,u}-{x}_{j,u}\right)}^2}, \end{equation*}

where ${O}_{ij}$ denotes the set of nonmissing proteins shared by samples *i* and *j*, *m* is the total number of proteins in the data matrix.

The Gaussian kernel function is then applied to convert the distance $d\left(i,j,X\right)$ between sample *i* and *j* to similarity:


(5) \begin{equation*} s\left(i,j\right)=\exp \left(-\frac{d{{\left(i,j,X\right)}_{\mathrm{Euclidean}}}^2}{2{\sigma}^2}\right)\kern-2pt, \end{equation*}

where *σ* controls the attenuation slowdown of similarity, and was set as the distance between sample *i* and its *k*-nearest neighbor (*k*NN) [19].

We denote ${y}_{i,u}$ as the weighted expression value of protein *u* in the *k* nearest samples of the sample *i*:


(6) \begin{equation*} {y}_{i,u}=\frac{\sum_{j\in N(i)}s\left(i,j\right){x}_{j,u}}{\sum_{j\in N(i)}s\left(i,j\right)}, \end{equation*}

where *N(i)* is the *k* NN samples found by the sample distance. ${x}_{i,u}$ was set to 0 if it is originally a missing value ${x}_{i,u}=\phi$. Because missing values in neighboring samples are encoded as zero when calculating ${y}_{i,u}$, the score jointly reflects local protein abundance and comissingness. A neighborhood containing repeated NAs produces a low ${y}_{i,u}$ supporting classification as a biological NA, whereas consistently detected and highly expressed neighbors produce a high ${y}_{i,u}$, supporting classification as a technical NA.

Finally, we assign a label ${l}_{i,u}$ of “biological” (${\phi}^b$) or “technical” (${\phi}^t$) to each weighted expression ${y}_{i,u}$ with a manual threshold *γ*:


(7) \begin{equation*} {l}_{i,u}=\left\{\begin{array}{@{}c}{\phi}^b,\kern0.5em if\ {x}_{i,u}=\phi, {y}_{i,u}\le \gamma \\{}{\phi}^t,\kern0.5em if\ {x}_{i,u}=\phi, {y}_{i,u}>\gamma \end{array}\right. \end{equation*}

Since technical NAs indicate that a value should have been present but was not detected, we restored technical NAs to 1 (let ${\phi}^t=1$) while keeping biological NAs as 0 (let ${\phi}^b=0$) in the binarized data matrix $\hat{X}$. The refined binarized data matrix $\hat{X}$ will be utilized for downstream biological tasks.

#### Downstream task 1: sample dimensionality reduction and clustering

For the refined binary data matrix $\hat{X}$, we employ Multidimensional Scaling (MDS) dimensionality reduction [20] based on recalculated Jaccard distance to project the data onto a two-dimensional space, facilitating subsequent processing and visualization.


(8) \begin{equation*} d{\left(i,j,\hat{X}\right)}_{\mathrm{Jaccard}}=\frac{m-{\sum}_{u=1}^m\delta \left({\hat{x}}_{i,u},{\hat{x}}_{j,u}\right)}{m-{\sum}_{u=1}^m\delta \left({\hat{x}}_{i,u},0\right)\bullet \delta \left({\hat{x}}_{j,u},0\right)\kern0.75em }. \end{equation*}

For benchmark datasets with known biological sample categories, *k*-means clustering was performed on the reduced-dimensional coordinate matrix *Z*, with the number of clusters *k* prespecified to match the number of known sample categories.

#### Downstream task 2: marker identification

To address the differences in protein presence and absence across distinct groups within the dataset, we developed the following methodology to identify characteristic biomarkers for specific groups [21].

For a dataset that contains sample groups A and B, the number of a specific protein *u* present in group A is ${n}_{\mathrm{A},1}=\sum_{i\in \mathrm{A}}\left({\hat{x}}_{i,u}=1\right)$, the number of its absence in group A is ${n}_{\mathrm{A},0}=\sum_{i\in \mathrm{A}}\left({\hat{x}}_{i,u}=0\right)$. The number of its present in group B is ${n}_{\mathrm{B},1}=\sum_{i\in \mathrm{B}}\left({\hat{x}}_{i,u}=1\right)$, the number of its absences in group B is ${n}_{\mathrm{B},0}=\sum_{i\in \mathrm{B}}\left({\hat{x}}_{i,u}=0\right)$. The log-transformed fold change of the protein in Group A relative to Group B is defined as


(9) \begin{equation*} {\log}_2\mathrm{FC}={\log}_2\left(\frac{n_{\mathrm{A},1}}{n_{\mathrm{A},1}+{n}_{\mathrm{A},0}}/\frac{n_{\mathrm{B},1}}{n_{\mathrm{B},1}+{n}_{\mathrm{B},0}}\right) \end{equation*}

Statistical significance is assessed according to the sampling design. For independent samples, a Pearson chi-square test with one degree of freedom is used, whereas for paired samples, McNemar’s test is applied to account for within-pair dependence. The groups are designated as A and B, with attributes valued as 1 and 0. The independent chi-square statistic is constructed as


(10) \begin{equation*} {\chi}^2=\frac{{\left({n}_{\mathrm{A},1}{n}_{\mathrm{B},0}-{n}_{\mathrm{A},0}{n}_{\mathrm{B},1}\right)}^2n}{\left({n}_{\mathrm{A},1}+{n}_{\mathrm{A},0}\right)\left({n}_{\mathrm{B},1}+{n}_{\mathrm{B},0}\right)\left({n}_{\mathrm{A},1}+{n}_{\mathrm{B},1}\right)\left({n}_{\mathrm{A},0}+{n}_{\mathrm{B},0}\right)}, \end{equation*}

where *n* is the total sample number.

For paired samples, where each sample in Group A is matched to a corresponding sample in Group B, the dependency within sample pairs is taken into account using McNemar’s test. For a specific protein *u*, the paired presence/absence outcomes are summarized as ${n}_{11}$, ${n}_{10}$, ${n}_{01}$, and ${n}_{00}$, representing the numbers of sample pairs with protein *u* present in both groups, present only in Group A, present only in Group B, and absent in both groups, respectively. McNemar’s test evaluates whether the two discordant outcomes occur with equal probability. The test statistic is calculated as


(11) \begin{equation*} {\chi}_M^2=\frac{{\left({n}_{10}-{n}_{01}\right)}^2}{\left({n}_{10}+{n}_{01}\right)}, \end{equation*}

which approximately follows a chi-square distribution with one degree of freedom under the null hypothesis.

For both independent and paired analyses, the log-transformed fold change is calculated from the marginal protein presence frequencies in Groups A and B as defined above. The resulting *P*-values are adjusted for multiple testing using the Benjamini–Hochberg procedure [22]. Markers for Groups A and B are identified by applying thresholds to the adjusted *P*-values and Log_2_FC.

#### Downstream task 3: PPI analysis

To achieve a more sensitive analysis of protein interactions, we consider the pattern of NAs in the two proteins when calculating the correlation coefficients. The Spearman correlation coefficients of the two proteins *u* and *v* in the dataset were first calculated [23, 24].

We introduce a weighted correlation *ρ* based on ${\rho}_{\mathrm{SCC}}$ with a reward-penalty weighting scheme


(12) \begin{equation*} \rho ={\rho}_{\mathrm{SCC}}\cdotp \left(1+{R}_{\phi}\right)\cdotp \left(1-{P}_{\phi}\right)\cdotp \left(1+{R}_{\phi^b}\right) \end{equation*}

The ${R}_{\phi }$ term rewards co-occurring *ϕ*s, ${P}_{\phi }$ penalizes mismatched patterns, and ${R}_{\phi^b}$ provides an additional reward for “biological NA” ${\phi}^b$s based on Equation (7)


(13) \begin{equation*} {R}_{\phi }=\alpha \sum_{i=1}^n\delta \left({x}_{i,u},\phi \right)\bullet \delta \left({x}_{i,v},\phi \right)\kern0.75em \end{equation*}

(14) \begin{equation*} {P}_{\phi }=\beta \sum_{i=1}^n\left(1-\delta \left({\overline{x}}_{i,u},{\overline{x}}_{i,v}\right)\right) \end{equation*}

(15) \begin{equation*} {R}_{\phi^b}=\alpha \sum_{i=1}^n\delta \left({l}_{i,u},{\phi}^b\right)\bullet \delta \left({l}_{i,v},{\phi}^b\right) \end{equation*}

Here, *α* and *β* are user-defined reward and penalty coefficients, respectively, with default values of $\alpha =\beta =0.01$. Finally, the *ρ* is adjusted to ${\rho}_{\mathrm{BIND}}$ in the range − 1 to 1:


(16) \begin{equation*} {\rho}_{\mathrm{BIND}}=\left\{\begin{array}{@{}c}\min \left(\rho, 1\right)\kern1.5em \rho >0\\{}\max \left(\rho, -1\right)\kern0.75em \rho \le 0\end{array}\right. \end{equation*}

With ${\rho}_{\mathrm{BIND}}$, a more sensitive analysis of protein interactions can be realized. We demonstrate this from several perspectives in the Results section.

### Simulation data

To evaluate the performance of classification algorithms in handling missing values, both “biological” and “technical” NAs must be simulated on a complete dataset. Since the actual categories of NAs in real datasets cannot be known, we employ the following controlled procedure to simulate both types of missing data on the complete dataset. The method is optimized based on previously reported proteomics approaches for simulating NAs [7]. The specific steps are as follows.

#### Prerequisites and definitions

Given a dataset *D* with NAs, remove all rows and columns that contain NA and obtain the resulting complete dataset ${D}_{\mathrm{full}}$, which is an *N× M* matrix containing *N* samples and *M* proteins, with all entries being known values. Let *a%* denote the target proportion of “biological” NAs relative to the total number of data points, and *b%* denote the target proportion of “technical” NAs. The combined proportion of the two types of NA ($a\%+b\%$) is set to match the total proportion of missing values in the original data matrix *D*.

#### Simulation of “biological” NAs

First, sort all *N× M* data points in ascending order based on the protein expression value. From the lowest $1.2\times a\%$ of data points in this sorted list, randomly select and mark *a%* of the total data points as “biological NA.” This ensures such missing events are preferentially introduced in low-value regions. Replace the values of the selected points with a NA and record their missing type.

#### Simulation of “technical” NAs

After the previous step, from all remaining nonmissing data points, randomly select and mark *b%* of the total data points as “technical NA.” Replace the values of these points with NA and record the type of each.

#### Outcome and evaluation

After these steps, a total of ($a\%+b\%$) data points in the matrix are set as missing, each with an explicit type label (biological/technical). The resulting dataset is used to evaluate NA-classification performance whose objective is to correctly predict the type of each missing value. By varying the parameters *a* and *b*, the impact of missing proportion and structure on algorithm performance can be assessed.

### Hyperparameter selection

When using BIND in practice, it is necessary to determine the hyperparameters *k* and *γ* due to differences in the data distributions across different datasets. First, preliminary hyperparameters should be determined using simulated data. Specifically, the simulation process described above should be used to evaluate candidate values of *k* and *γ*, and appropriate parameters should be selected based on NA classification performance, while also considering computational efficiency. In the method evaluation in this paper, the parameter ultimately used was the near-optimal option *k* = 10 and *γ* = 3. For the conventional *k*NN-imputation baseline, the number of nearest neighbors was set to *k*_*k*NN-Imp_ = 10. Since the simulation itself cannot fully capture the characteristics of real biological data, appropriate hyperparameters should additionally be validated through their performance in downstream biological tasks (such as clustering and biomarker identification).

### Public data processing and experimental details

We used three human cancer cell line datasets [28–30], one human liver cancer dataset [31], and one single-cell proteomics (SCP) dataset [32]. We benchmarked a variety of commonly used imputation methods, including single-variable methods (MinDet, MinProb), global methods (Singular Value Decomposition (SVD), Bidirectional Principal Component Analysis (BPCA)), local methods (*k*NN, missForest), and deep learning-based methods (Proteomics Imputation Modeling Mass Spectrometry, (PIMMS)) [13]. For a detailed description of the experimental procedure, see Supplementary Methods and Supplementary Table S1.

### Bioinformatics tool and web server implementation

The algorithm and function of BIND were compiled in R (version 4.5.3). The web tool was implemented based on JavaScript. Users can access and process their data freely in BIND without any login requirement or cookies through some popular web browsers. Users can choose to operate this tool on their computers by using our provided command-line scripts. The detailed user manual can be found in our GitHub repository (see Data availability).

## Results

### Overview

The whole pipeline operates in three modules (Fig. 1): (i) NA statistics and visualization. Data matrices were binarized (1 = detected, 0 = NA). We quantify the proportion of NAs for each protein and visualize their distribution patterns using histograms, pie charts, etc. Specific patterns of missingness were identified within each sample group based on sample grouping. (ii) NA classification. Using adaptive *k*NN with Gaussian kernels, BIND integrates neighborhood expression and NA co-occurrence to classify each NA as biological or technical (see Methods for details). (iii) Downstream tasks. After classification, technical NAs are restored to presence in the binary matrix and biological NAs remain 0. The dataset was then applied to downstream biological tasks: sample dimensionality reduction, clustering, and grouping, identification of signature biomarkers, and missing-value-weighted Spearman correlation analysis. Finally, we established a user-friendly interactive web server for BIND, providing researchers unfamiliar with coding with convenient analytical tools.

### NA patterns in proteomics data are biologically informative

First, we demonstrate the biological significance of missing values using human cancer cell line datasets (CCLE949, NCI60, and CRC65) [28–30]. These datasets are also subsequently employed to evaluate the BIND method. Binary heatmaps illustrated the global pattern of NAs (Fig. 2A, Supplementary Fig. S1). Overall, CCLE949 contains 6692 proteins, and 487 proteins (7.3%) were detected in all 949 samples (no NA). In addition, 1808 (27%), 2337 (34.9%), and 2060 (30.8%) proteins were recorded as NA in <20%, 20%–80%, and >80% of samples, respectively (Fig. 2B). To measure the magnitude of missingness, we used the “NA proportion” to represent the frequency at which a particular protein was recorded as “NA” in the dataset. Histograms indicated that the datasets have more proteins with high and low NA proportions than in the middle range (Fig. 2C, Supplementary Fig. S2). These three datasets exhibit different detection depths (Fig. 2D), specifically 6692(CCLE949), 9709(NCI60), and 11 241(CRC65). CCLE949 had the lowest identification depth, and it also had the highest proportion of proteins with an NA proportion >0.8 (30.8%) (Fig. 2E); this may be because it includes various types of tissues. The CRC65 dataset had the highest identification depth, and it also had the highest proportion of proteins with an NA proportion of 0 (41.9%); this may be because the dataset consists solely of colorectal cancer tissues, representing a single source. Notably, the protein NA proportions displayed a significant negative correlation with the mean expression levels of the corresponding proteins (*r* = −0.55, *P* < 2.2e−16) (Fig. 2F). This was also observed among individual samples in which the high-abundance proteins tend to have low NA proportions (Supplementary Fig. S3). This suggests that the NA frequency could reflect the abundance of the protein.

**Figure 2:**
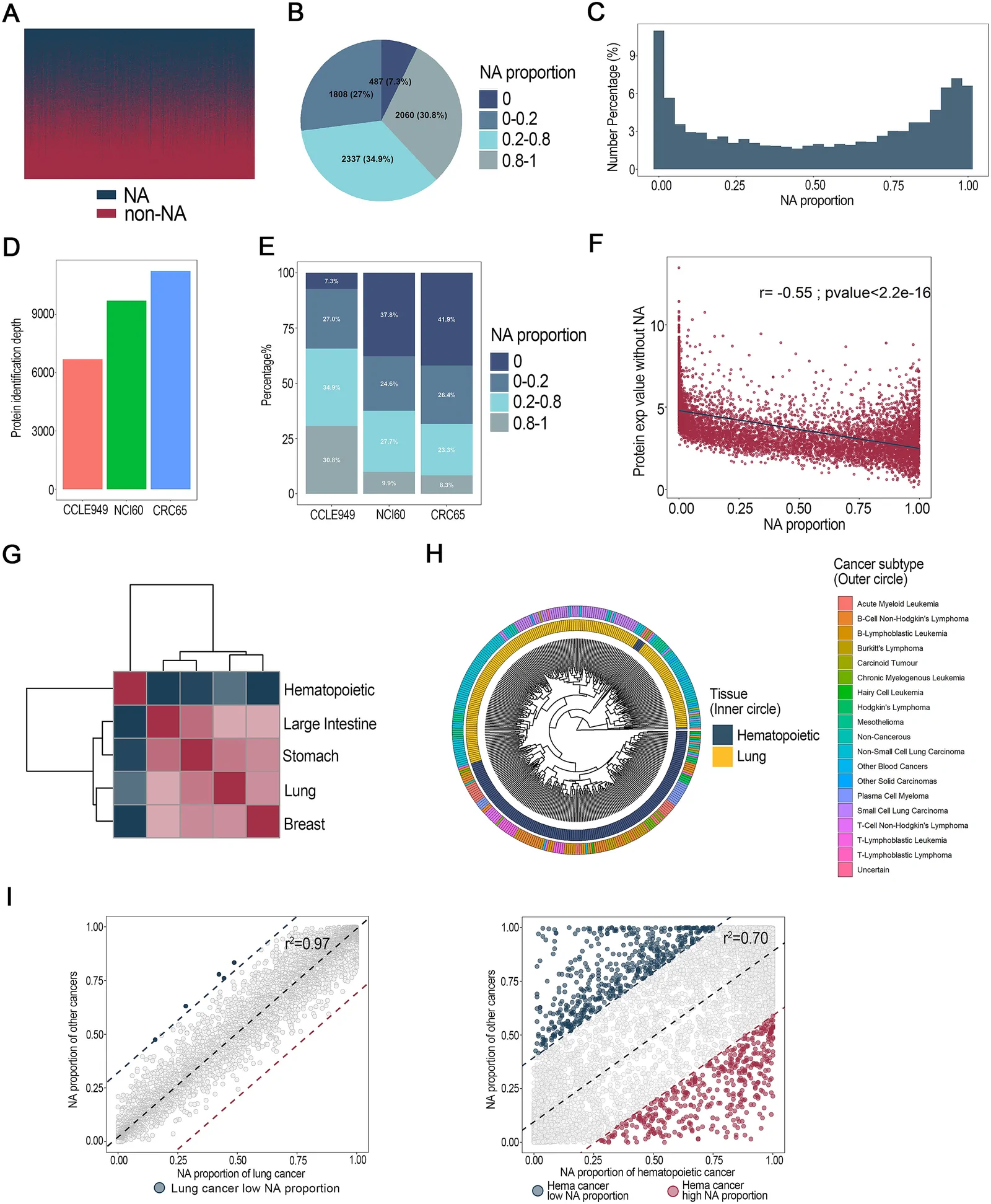
Missing value patterns in proteomics carry biological significance. (A) Binarized heatmap of missing values for human cancer cell line datasets CCLE949. (B) NA distribution pie chart. (C) NA proportion distribution histogram of the dataset. (D) Protein identification depth in CCLE949, NCI60, and CRC65 dataset. (E) NA proportion composition across the three datasets. (F) Scatter plot of mean expression value to NA proportion of proteins. (G) Correlation heatmap of NA proportions in hematopoietic, large intestine, stomach, lung, and breast tissue. (H) Sample clustering using Jaccard distance for the binarized data. The result shows an excellent separation between hematopoietic cancer and lung cancer. (I) Scatter plot of NA proportions in other tissues to NA proportions in lung cancer. Lung cancer had a similar NA pattern to other tissues (left). Scatter plot of NA proportions in other tissues to NA proportions in hematopoietic tissue. Hematopoietic cancer showed significant differences from other tissues (right).

The cell lines in CCLE949 were derived from various tissues (Supplementary Fig. S4). We investigated the NA patterns of the hematopoietic, lung, breast, large intestine, and stomach cancer cell lines. Clustering analysis based on the NA proportions revealed that the solid cancers are highly correlated in their NA patterns but are distinct from the hematopoietic cancers (Fig. 2G). To determine whether NA could serve as a specific feature that classifies tissue types/subtypes, we binarized the dataset by assigning “0” to NAs and “1” to the detected values. We performed clustering analysis of the hematopoietic and lung cancer samples with Jaccard distance, which is tailored for binary data [33]. The results showed excellent separation of the two cancer types with subtype stratification of the lung cancer cell lines (Fig. 2H). Interestingly, NA-based binary clustering produced a better outcome than clustering with expression values after removing all NAs (Supplementary Fig. S5). This result illustrates that NAs could inform the specific absence of proteins and differentiate tissue types.

To further investigate the NA features of the two cancer types, we compared the NA proportion of their proteomes with other cancer types (Fig. 2I). Scatter plots revealed a good correlation between the NA proportions of the lung cancer proteome and the other types of solid tumor (Fig. 2I left panel). In contrast, the hematopoietic cancers displayed a poor correlation in protein NA proportions with the other cancer types (Fig. 2I right panel). Gene Ontology (GO) enrichment analysis showed that proteins with high NA proportions in hematopoietic cancers were enriched in focal adhesion and cell junction assembly (Supplementary Fig. S6). This is consistent with the absence of such structures in hematopoietic cell lines, which are typically maintained in suspension culture. In contrast, proteins with low NA proportions in hematopoietic cancers were mainly involved in immune cell activation and immune response signaling, consistent with their absence from the solid-tumor cell lines (Supplementary Fig. S7). Our results indicate that NA patterns in cell line proteomes can discern the tissue-specific presence or absence of proteins.

### BIND deconvolves the nature of NA and improves sample clustering

In addition to representing low/no expression, NAs can also be due to experimental errors. To excavate the biological information carried by the missing values, it is important to deconvolve the nature of NAs, which we classify here into two categories: “biological” NAs that reflect no/low protein expression, and “technical” NAs introduced by nonbiological errors. We use a NA deconvolution algorithm that applies an adaptive *k*NN-based approach to determine the nature of NAs (Fig. 1B; see Methods for details) [34–36]. Briefly, BIND dynamically adjusts the Gaussian kernel bandwidth for each sample based on the local neighborhood structure to compute context-aware similarity weights [37]. These weights are then integrated with simultaneously captured expression values and co-occurrences of missing values within the neighbors. Finally, BIND determines “biological” or “technical” NAs that are below or above an optimized threshold *γ*, respectively. The selection of hyperparameters *k* and *γ* should be based on both simulation data and downstream biological tasks (see Methods for details).

We exploited the CCLE949 dataset and generated a collection of simulated “biological” and “technical” NAs to optimize BIND. Briefly, a fraction of low-expression proteins was removed to create “biological” NAs, and an additional fraction of quantified proteins was discarded to mimic “technical” NAs. Reiterative modeling demonstrated that the performance of BIND improves with increasing *k* (number of neighboring samples) and varying threshold *γ* (Fig. 3A). For the simulated dataset, we selected *k* = 10 and *γ* = 3 as near-optimal parameters considering both NA classification performance and computational efficiency, achieving an AUC of 0.9282 in deconvolving the two types of NAs (Fig. 3B, Supplementary Fig. S8).

**Figure 3:**
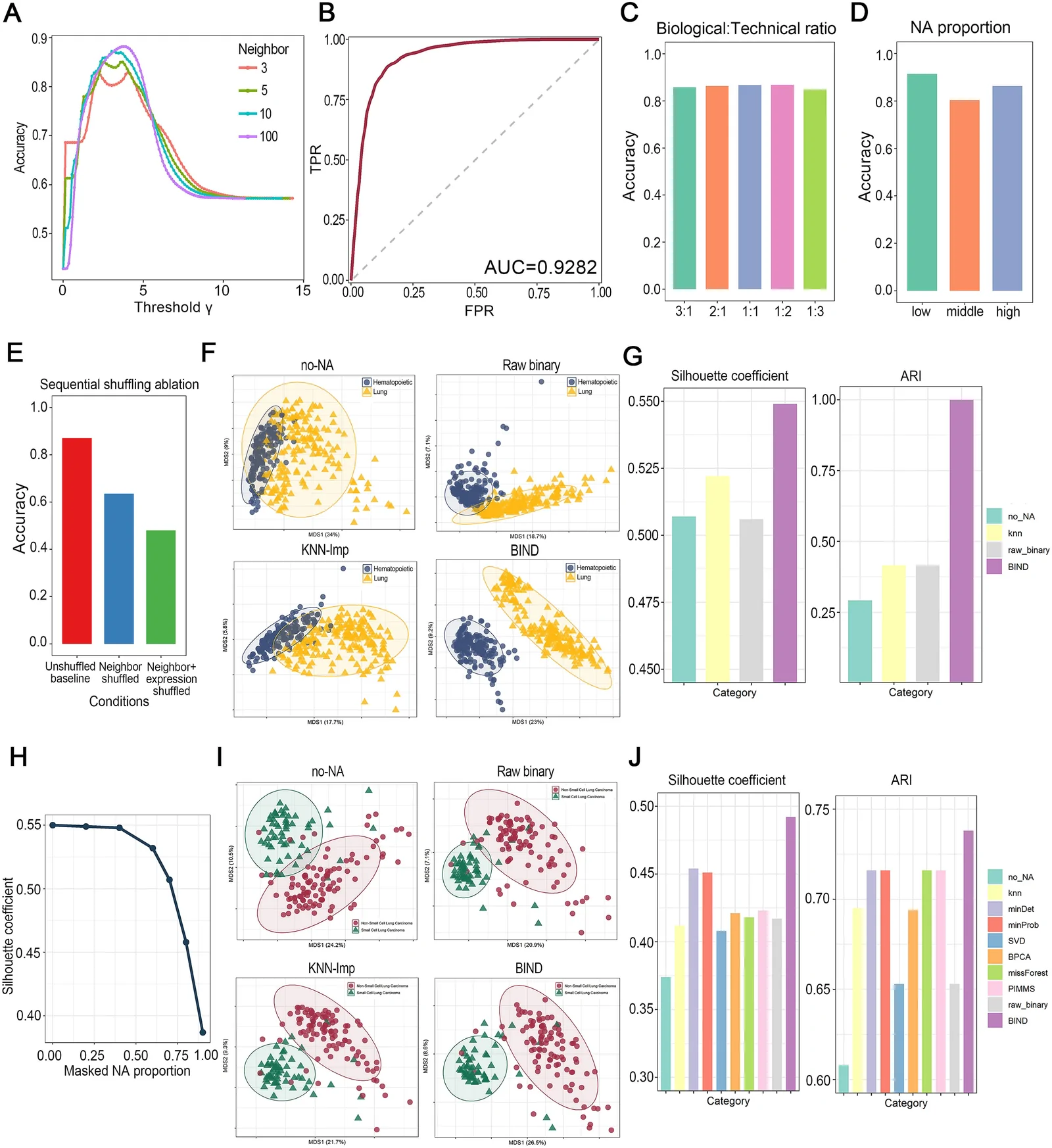
BIND classification performance and sample clustering performance evaluation. (A) Variation of NA classification accuracy with different parameters in the simulation dataset, we chose *k* = 10 and threshold =3 for further study. (B) When *k* = 10, the AUC of NA classification achieved at 0.9282. (C) The proportions of biological vs. technical NA in the simulated dataset had no effect on classification accuracy results. (D) Accuracy of NA classification for proteins with different NA proportions. (E) NA-classification accuracy under sequential shuffling ablation. The three conditions represent the unshuffled baseline, shuffled neighborhood topology, and shuffled neighborhood topology with additional shuffling of expression values within the randomized neighborhoods, respectively. Expression shuffling was performed after neighborhood shuffling. (F) MDS dimensionality reduction and k-means clustering results for all non-NA data in the dataset (no-NA), the results of *k*NN imputed data (KNN-Imp), the results of binarized raw data (raw binary), and BIND-processed data (BIND). (G) Silhouette coefficient and ARI of sample classification for the four types of data. BIND achieved the best performance. (H) Silhouette coefficient results after masking proteins with a proportion less than the corresponding NA proportion. When proteins with an NA proportion of 50% or higher were masked, the clustering performance declined most significantly. This indicates that BIND effectively utilizes information from proteins with high missing rates. (I) MDS dimensionality reduction and k-means clustering results of SCLC/NSCLC data. (J) Comparison of the silhouette coefficient and ARI results from various imputation methods with those from BIND.

To test the robustness of the method, we also adjusted the ratio of biological NA to technical NA in the simulated data. We found that within the range of 3:1‑1:3, classification accuracy remained relatively stable across the evaluated biological-to-technical NA ratio (Fig. 3C). To analyze the effect of NA proportion on NA classification, we divided the proteins in the dataset into three categories based on their NA proportion: low (0–0.4), middle (0.4–0.6), and high (0.6–1). In simulated datasets, BIND achieved classification accuracies of 0.914, 0.804, and 0.863, respectively, indicating robust performance across different NA levels (Fig. 3D). Furthermore, sequential shuffling ablation (Fig. 3E) showed that BIND uses both neighborhood topology and local expression. Accuracy decreased from 0.871 at baseline to 0.635 after neighborhood topology shuffling and to 0.480 after additional expression value shuffling, indicating that neighborhood information is critical for BIND’s effectiveness.

Since “technical NA” indicates a nondetected value, we restored it to 1 in the binary data, while maintaining “biological NA” at 0. The resulting BIND datasets were evaluated in several downstream analyses, including sample clustering, biomarker identification, and PPI) analysis (Fig. 1C).

For sample clustering, we compared the binary BIND data with the data matrices after removing all proteins with NA values (no-NA), the data matrices after conventional *k*NN imputation (KNN-Imp), the data matrices after binarization (raw binary). We utilized MDS dimensionality reduction on the data matrices. After dimensionality reduction, the data were clustered into two categories using *k*-means. The effectiveness of the approach was evaluated based on the silhouette coefficient and adjusted Rand index (ARI) of the results [25, 26]. Figure 3F shows the results of dimensionality reduction and clustering for the four data matrices (no-NA, KNN-Imp, Raw binary, and BIND). Their silhouette coefficients were 0.50, 0.52, 0.51, and 0.55, respectively. The ARI values were 0.29, 0.42, 0.42, and 1, respectively (Fig. 3G). We tested the sensitivity of *k*NN imputation to hyperparameters and found that the number of neighbors has little effect on clustering performance (Supplementary Fig. S9). This demonstrates the powerful capabilities of BIND in sample clustering and grouping. Next, we examined the effect of the NA proportion on sample clustering using BIND. We masked the BIND-processed binary data matrix based on the NA proportion of each protein, specifically by setting the value to 1 for all proteins with an NA proportion < *n*%. The value of *n* ranges from 10 to 90. We found that when the proportion of masked NA was less than 50%, the silhouette coefficient decreased only slightly, whereas it decreased significantly in the 50%–90% range (Fig. 3H). This suggests that proteins with 50%–90% NA contain a substantial amount of information capable of identifying the sample. Since proteins with a high NA proportion are currently typically removed during data preprocessing, we believe that BIND preserves information that is missed by traditional imputation methods.

To further validate BIND’s performance and reliability, we split the lung cancer data into small-cell lung cancer (SCLC) and non-small-cell lung cancer (NSCLC) to evaluate the clustering results. MDS dimensionality reduction demonstrated that BIND outperforms no-NA, KNN-imp, and raw binary data (Fig. 3I). Additionally, we compared various commonly used proteomics data imputation methods, including the single-value approaches MinDet and MinProb, the global methods SVD and BPCA, the local methods *k*NN and missForest, and the self-supervised deep learning-based imputation method, PIMMS (Fig. 3J, Supplementary Fig. S10). BIND achieved the best silhouette coefficient and ARI, at 0.492 and 0.738, respectively.

Overall, this suggests that in the process of multivariate analysis of proteomics data, BIND is able to alleviate interference from “technical” noise and achieve superior sample clustering results.

### BIND restores biomarkers and PPIs overlooked by traditional methods

For biomarker identification, we calculated fold changes based on the ratio of protein presence in two groups and employed chi-square tests for significance assessment. This approach identified markers for lung cancer and hematopoietic cancer (Fig. 4A). We also performed *k*NN imputation (KNN-Imp) and differential expression analysis on the data to compare the ability of traditional methods to identify biomarkers with that of BIND. Among the biomarkers identified by BIND, only 14.8% and 17.1% of those in hematopoietic cancer and lung cancer, respectively, were also identified by KNN-Imp (Fig. 4B). This demonstrates BIND’s potential for discovering new biomarkers.

**Figure 4:**
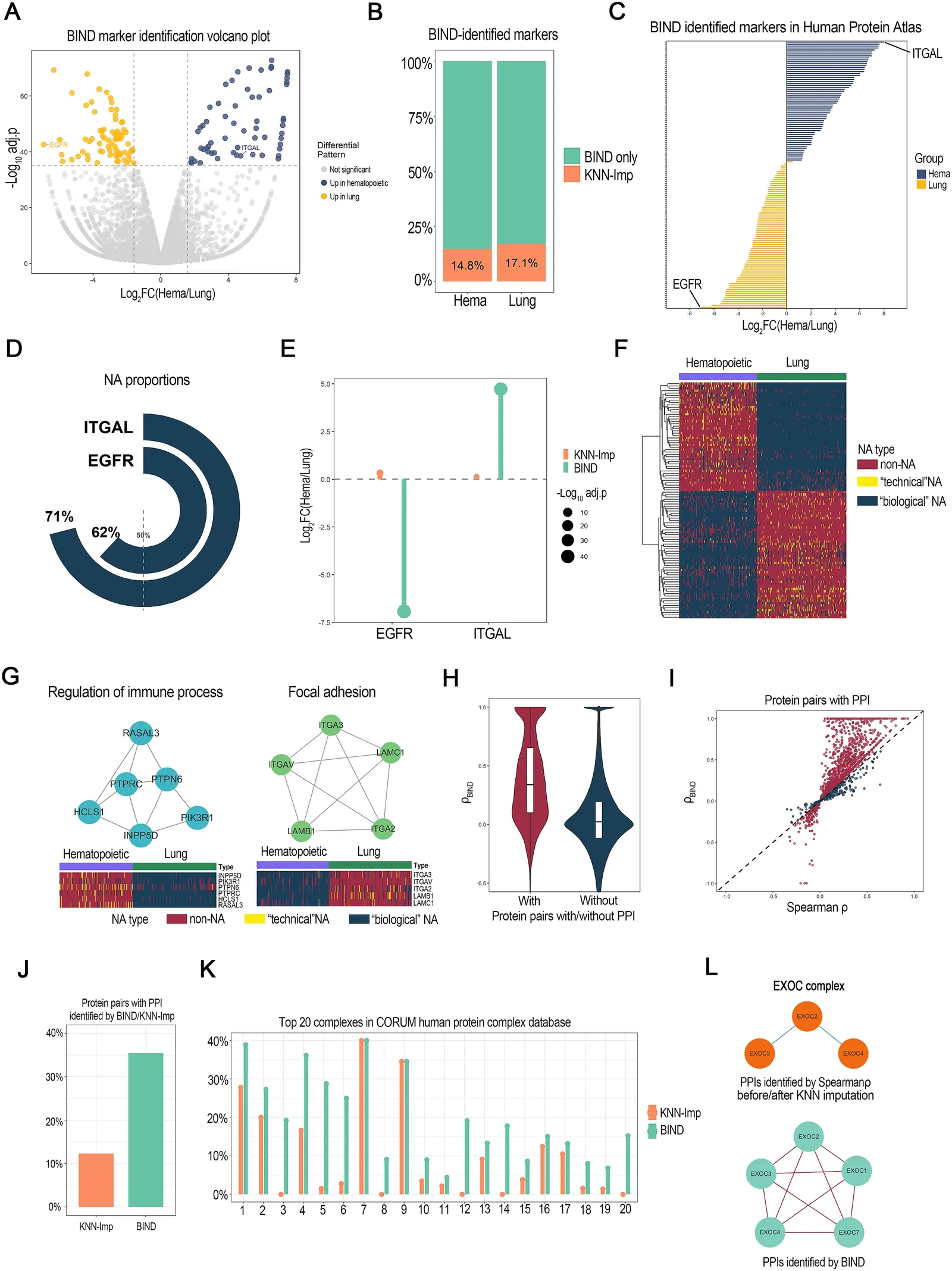
BIND identified biomarkers and PPIs undetectable by imputation methods. (A) Volcano plot of BIND-identified hematopoietic cancer and lung cancer biomarkers. (B) The percentage detected by *k*NN imputation among the markers identified by BIND. (C) Fold change of markers identified by BIND in the hema/lung tissues within the Human Protein Atlas database. ITGAL and EGFR were the markers showing the greatest differences in blood and lung cancer tissue. (D) NA proportions of ITGAL and EGFR in the dataset. (E) Log2 fold changes and *P*-values for ITGAL and EGFR identified by BIND and KNN-Imp. KNN-Imp was unable to identify these two biomarkers, despite their being widely described as highly expressed proteins in blood and lung cancer. (F) Heatmap of BIND identified hematopoietic cancer and lung cancer biomarkers. (G) BIND helps to recover proteins that are exclusive to hematopoietic cancer. This protein complex is involved in the immune process pathway(left). BIND helps restore proteins that are unique to lung tissues. This protein complex is involved in focal adhesion pathways (right). (H) Distribution and mean value of *ρ*_BIND_ in protein pairs with and without PPIs. (I) Scatter plot of protein pairs with PPI, *ρ*_BIND_ to Spearman’s *ρ*. (J) The percentage of known PPIs identified by KNN-Imp and BIND, respectively. (K) The proportion of protein pairs identified as PPIs by KNN-Imp and BIND among the top 20 complexes in the CORUM protein complex database. (L) PPI in EXOC protein complex identified by spearman *ρ* and *ρ*_BIND_, 0.5 as threshold. *ρ*_BIND_ identified all 10 interactions.

To verify whether the biomarkers identified by BIND are biologically reliable, we compared the hematopoietic cancer/lung cancer fold changes for these biomarkers across tissue-derived cell lines in The Human Protein Atlas (HPA) database (Fig. 4C). All hematopoietic cancer markers identified by BIND exhibited a high hematopoietic-to-lung cancer fold changes in the HPA database. Similarly, nearly all lung cancer markers identified by BIND exhibited a low fold change in the HPA database (with only one exception). In the HPA database, the biomarkers showing the greatest differences between hematopoietic and lung cancer were integrin alpha L (ITGAL) and Epidermal Growth Factor Receptor (EGFR), respectively. The proportions of NA values for ITGAL and EGFR in the original dataset were 71% and 62%, respectively (Fig. 4D). This suggests that they are highly likely to be overlooked by traditional data processing methods but can be detected by BIND—particularly EGFR, which is a well-known lung cancer biomarker. Even if we loosen the threshold for imputation so that both proteins underwent *k*NN imputation and differential analysis, we were still unable to identify them as statistically significant biomarkers (Fig. 4E).

We used a clustering heatmap to display the biomarkers identified by BIND and labeled the proteins that BIND identified as “technical NA,” indicating that they should have been present but were not detected (Fig. 4F). For example, a large number of NAs of HCLS1, PTPRC, INPP5D, PTPN6, RASL3, and PIK3R1 are determined as “technical” ones in hematopoietic cancer samples, but not in other cancers (Fig. 4G, left panel). This suggests the unique presence of these proteins in hematopoietic cells, in line with their immune-regulation functions. In contrast, ITGA2, ITGA3, ITGAV, LAMB1, and LAMC1 have most NAs determined as “biological” ones in hematopoietic samples and as “technical” ones in other cancer types (Fig. 4G, right panel). This aligns well with their functional implications in the focal adhesion pathway, which are insignificant in hematopoietic samples. Interestingly, these proteins have intricate PPIs, which prompted us to investigate whether BIND could help improve PPI analyses.

Protein expression covariance has been exploited for PPI analysis. However, the presence of NAs has been a significant barrier for such analysis. We propose that coappearance as biological NAs is a rewarding factor when calculating expression covariance of two proteins. Therefore, following deconvolving biological NAs, BIND implements a missing value-weighted approach to generate *ρ*_BIND_, an NA-assisted correlation coefficient to improve PPI analysis (Fig. 1C, see Methods for details). We first analyzed the hematopoietic cancer samples in the CCLE949 dataset. Human protein interactions were obtained from the STRING database [27], and 3556 pairs of physical interactions with PPI scores > 0.9 were considered “with PPI.” The mean value of *ρ*_BIND_ for protein pairs with PPI was higher than that for protein pairs without PPI (Fig. 4H). For 79.1% of these PPI pairs, *ρ*_BIND_ yielded higher association coefficient values than the conventional Spearman’s *ρ* (Fig. 4I, Supplementary Fig. S11).

We also compared the PPI identifications made by BIND and KNN-Imp. Among known human PPI, KNN-Imp was able to identify only 12% of PPIs, while BIND identified 35% (Fig. 4J). Among the top 20 complexes in the CORUM human protein complex database, BIND identified more PPIs for 18 of them than KNN-Imp identified (Fig. 4K). Moreover, *ρ*_BIND_ established 10 (100%) interactions among the Exocyst (EXOC) complex, whole conventional Spearman’s *ρ* only detected 2 (20%) interactions (Fig. 4L). This is also the case for Mediator (MED) and Syntaxin (STX) (Supplementary Fig. S12), and others. In summary, *ρ*_BIND_ could further extract information from missing values, and improve the PPI analysis with expression covariation.

### BIND identification of biomarkers and PPIs in human liver cancer tissues

We analyzed a proteomics dataset from human hepatocellular carcinoma (HCC) tissue and adjacent normal liver tissue [31]. This dataset comprises 248 samples, with 124 cancer samples and 124 normal tissue samples (Fig. 5A). Proteins with no NA, <0.2 NA, 0.2–0.8 NA, and >0.8 NA accounted for 13.4%, 28.8%, 33.1%, and 24.7% of the total 9145 proteins, respectively (Fig. 5B and C). BIND effectively separated cancer samples from normal samples in clustering analyses, indicating characteristic NA patterns (Fig. 5D).

**Figure 5:**
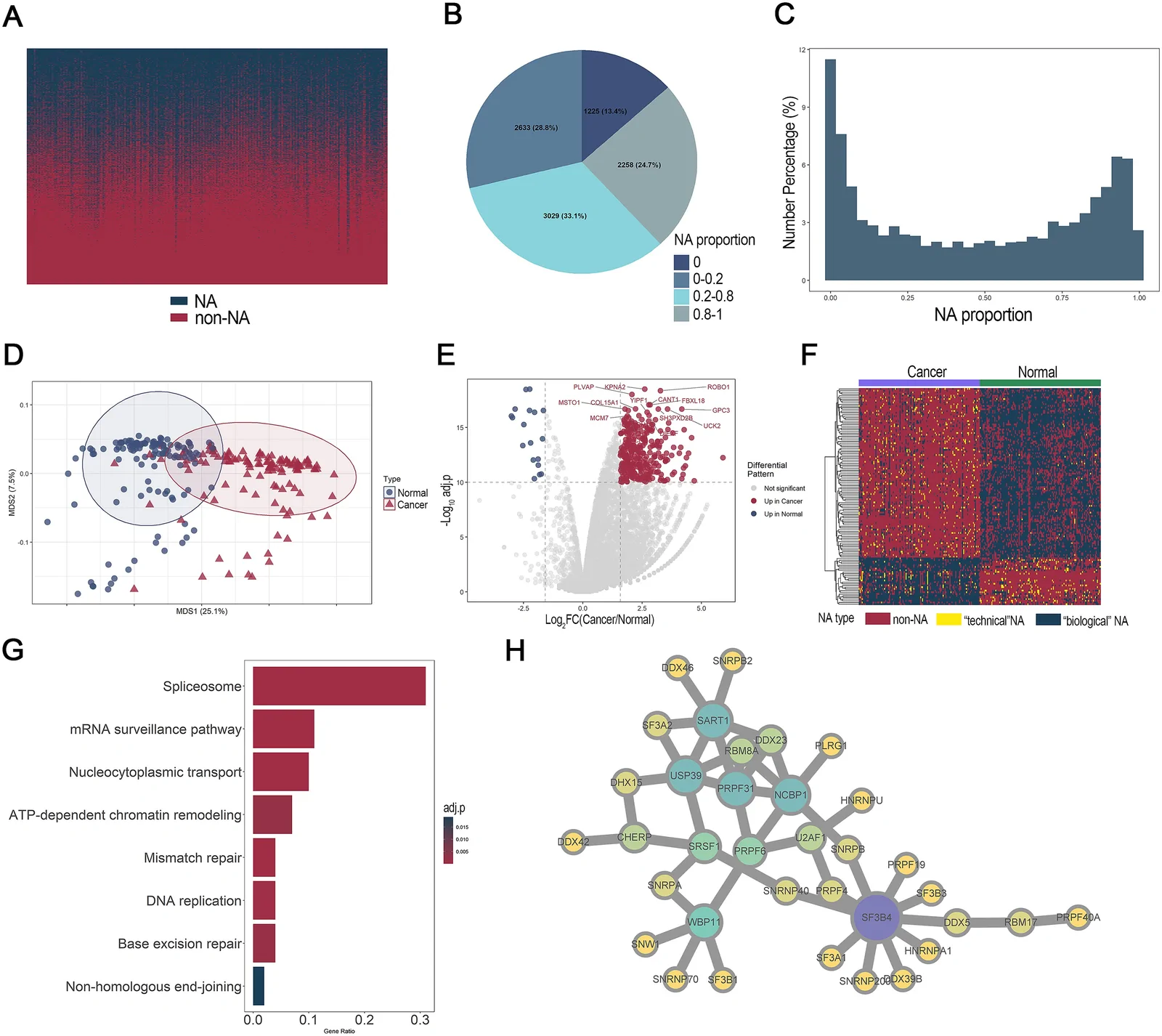
Application of BIND in human hepatocellular carcinoma clinical dataset (HCC). (A) Binarized heatmap of the dataset. (B) Pie chart of the NA proportions. The percentage of no-NA, <0.2, 0.2–0.8, and 0.8–1 NA proteins are 13.4%, 28.8%, 33.1%, and 24.7%. (C) Distribution histogram of HCC dataset. (D) MDS dimensionality reduction result of BIND processed data. Dimensionality reduction of binary datasets processed by BIND can distinguish cancerous and normal HCC tissues. (E) Volcano plot of HCC dataset. Proteins with |log2FC| > 1.5 and -log10(padj) > 10 were identified as group-specific markers. (F) Heatmap of top 100 markers of cancer tissue and normal tissue found by BIND. (G) Kyoto Encyclopedia of Genes and Genomes (KEGG) pathway enrichment analysis of proteins in cancer-specific PPIs found by BIND. (H) Cancer-specific PPIs in spliceosome pathway.

Since the HCC dataset consists of paired data, the McNemar’s test was used to identify markers. BIND also effectively improved biomarker identification for HCC (Fig. 5E and F). For example, GPC3 is a widely reported biomarker of HCC [38, 39], but it was NA in 65% of the samples and could be missed by traditional analyses. We have also identified several candidate HCC biomarkers, such as KPNA2 and ROBO1. Pathway enrichment analysis indicates that these HCC biomarkers are primarily associated with processes such as cell cycle and ribosome pathway (Supplementary Fig. S13). Through PPI analysis using BIND, we identified characteristic PPIs for HCC with a score >0.7 in cancer tissues and a difference >0.5 between cancer and normal tissues. These PPIs primarily involve proteins associated with pathways such as the spliceosome and DNA replication (Fig. 5G). Key proteins in the spliceosome complex include SF3B4, NCBP1, SART1, etc. (Fig. 5H). Our findings demonstrate that BIND can effectively identify biomarkers in clinical data, revealing that the spliceosome complex may play a significant role in HCC.

### A user-friendly BIND web server

Next, we compiled BIND into a web-based tool for researchers with varying computational skills to explore and process NAs in large proteomics datasets interactively. It executes the following functions: (i) Data input. A proteomics dataset containing missing values is imported with the grouping information. (ii) Characterization of missing value patterns in the dataset. This part analyzes the pattern of missing values in the dataset and the differences of missing values in different subgroups through various visualization methods, such as heatmaps, pie charts, etc. BIND analyzes the NA frequency of proteins and performs sample clustering based on the binary transformation of missing values. (iii) Classification of NAs. BIND differentiates “biological” from “technical” NAs by integrating features of neighboring samples (see Methods for details). This deconvolves the NA natures and helps interpret the biological significance. (iv) NA-weighted protein interaction analysis. BIND generates weighting factors based on biological NAs to enhance the abundance covariance analysis, thereby improving the sensitivity and precision of protein complex mapping.

The BIND website provides pages including home page, user tutorial, example data view, task submission, task query, and result view (Fig. 6A). The web page is compatible with various popular computer systems (Windows, Mac, Linux) and web browsers (Chrome, Edge, Firefox, Safari, etc.).

**Figure 6:**
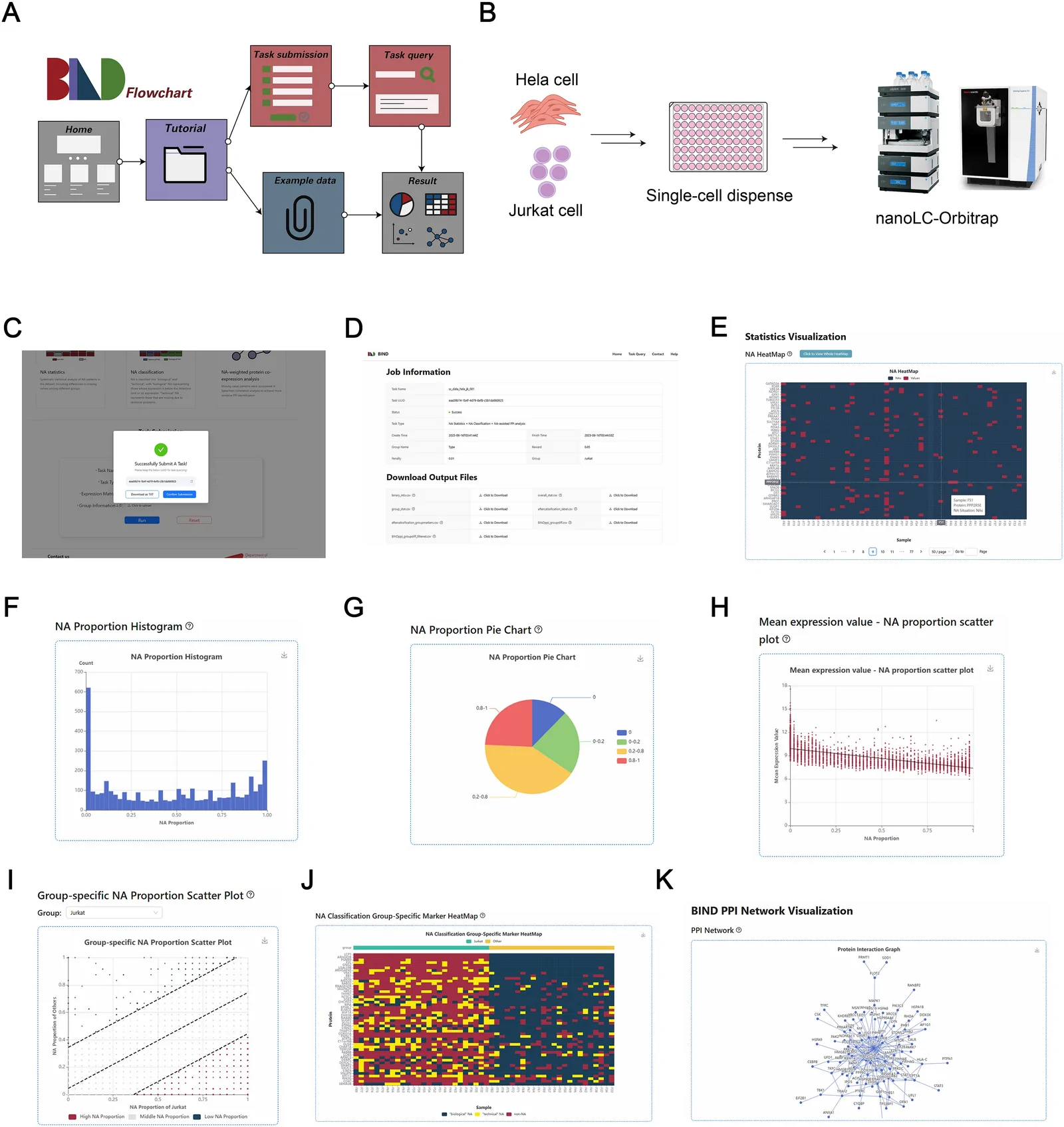
The online web server for BIND. (A) Flowchart of the BIND website. (B) SCP process of Hela and Jurkat cells. (C) Task submission page. (D) Task information page. After task completion, the user can view the result on an interactive page. (E) Binary heatmap showing NA and expression values. (F) Histogram of the distribution of NA proportion. (G) NA proportion’s pie chart. (H) Scatterplot of NA proportion vs. expression of this protein. The expression of the protein shows a negative correlation with the proportion of its missing. (I) Group comparison scatter plot of NA proportion (Hela vs. Jurkat). (J) The Jurkat cell protein marker heatmap discovered by BIND. (K) Unique PPIs for Jurkat cells in the regulation of immune response pathway identified by BIND.

### Analyzing SCP data on the BIND web server

SCP enables the analysis of protein expression at the individual cell level, providing insights into cellular heterogeneity that cannot be captured by bulk-scale studies. SCP has developed rapidly recently for understanding complex biological mechanisms. We expect that the deconvolution of NAs in the SCP datasets could provide additional insights. Therefore, we examined the operation of the BIND web server utilizing an SCP dataset of two cell lines (Fig. 6B) [32]. After task submission, a Universally Unique Identifier (UUID) is generated for task query (Fig. 6C). Upon completion, visualization/download of results can be performed on the task information page (Fig. 6D). The results include a heatmap of NA distribution (Fig. 6E) as well as histograms and pie charts of NA proportions (Fig. 6F and G). Similar to the analysis of bulk samples, we observed a negative correlation between NA proportion and expression of a protein (Fig. 6H). Selective analysis could reveal proteins that are uniquely missing/present in Jurkat cells relative to Hela cells (Fig. 6I). Following the NA deconvolution of SCP data, BIND identified protein markers of Jurkat cells (Fig. 6J), including lymphocyte cytosolic protein 1 and Enah/Vasp-like. These protein markers are implicated in pathways such as cell leading edge and phagocytic vesicle (Supplementary Fig. S14). Moreover, BIND improved PPI analysis and identified a GDP dissociation inhibitor 1-centered complex that is unique to Jurkat cells (Fig. 6K).

Overall, the BIND server represents a programming-free and interactive web-based tool for NA deconvolution. With the growing amount of large-scale proteomics and SCP data, it is expected to further our understanding of complex biological processes and disease mechanisms.

## Discussion

Technological advances have greatly improved the throughput and sensitivity of proteomics analyses, even to the single-cell level. Ironically, this typically leads to not fewer, but more missing values (NAs), which represent an immense challenge in processing large-scale proteomics datasets. Data imputation is commonly used to facilitate statistical analyses, assuming that the data matrix is necessarily complete. However, NAs could reflect biological absence/scarcity, and simply filling them may obscure the biological meanings. BIND provides a novel analytical framework for deconvolving proteomics data from the perspective of the NAs, aiming to excavate the underlying biological significance.

The presence or absence of specific proteins is a key feature of different biological samples. However, limited insights are available from examining the patterns of missing proteins in large-scale proteomics datasets. An early study identified unique proteins in patients with rheumatoid arthritis based on the proportion of missing data, but this approach has not been studied in greater depth [14]. Our results showed significant differences in the proportion of NAs in cancer cell lines of different tissue origins. Notably, the potential biological information could be mined after the binary transformation of NAs and expression values. This prompts us to develop BIND to excavate the biological significance of missing values.

Missing values are typically classified as MCAR, MAR, or MNAR [40], though these types are rarely distinguished in practice due to difficulty in identification. One recent method differentiates types by expression distribution but assigns a single type per protein, which is insufficient [41]. BIND classifies each missing value as “biological” or “technical” based on neighboring samples, unlike conventional imputation methods that overlook NA nature [42].

BIND is effective and highly interpretable, as it is based on characterizing the status of the concerned protein in neighboring samples. It defines “technical” NAs as the result of random technical issues, and considers it as a protein presence. In contrast, “biological” NAs are treated as a protein absence in the sample. This facilitates the identification of protein markers in different biological scenarios. In liver cancer, we identified the widely reported marker GPC3, as well as candidate markers such as KPNA2 [38, 39]. For PPI, the use of the PPI database to recover missing proteins in the dataset has been reported [43]. BIND takes the other direction and incorporates the pattern of biological absence into account in the correlation analysis, offering better robustness. BIND also identified EXOC, MED, and STX complexes in hematologic cancers by PPI analysis. EXOC and STX play a role in vesicle transport, while MED plays a role in the regulation of RNA polymerase II [44–46]. In liver cancer, BIND-identified spliceosome-associated protein complexes and PPIs related to the DNA replication pathway [47–49].

Finally, we provide the BIND web server for biology researchers unfamiliar with programming to perform data analysis. The web server has clean and clear pages and provides detailed instructions for operation. Its data analysis process is highly modular and highly interactive, allowing users to extract biological information of interest. We demonstrated the reliability of BIND for single-cell data analysis by testing an SCP dataset on this web server. BIND could robustly analyze SCP data, and identify characteristic protein markers as well as important PPI.

This study has some limitations. Real missingness can be influenced by factors such as batch effects, signal suppression, matrix interference, and peptide-specific biases, resulting in more complex patterns than those captured by our current model. Although the method used in this paper to simulate missing value mechanisms is consistent with existing literature, there is still a possibility that it may not accurately reflect the reality of proteomics data. Since there are currently very few ways to determine the “ground truth” of missing value types in a dataset, research into the mechanisms underlying missing values remains constrained by this issue. Future research will benefit from the continued development of simulation frameworks that can better model complex missing-value behavior at both the biological and instrumental levels.

In conclusion, BIND is distinct from the traditional missing value imputation process and provides unique insights into missing value-centered proteomics data analysis. We anticipate that BIND can facilitate in-depth analysis of proteomics data and help address the missing-value problem. We also hope that the concept of “extracting meanings from the missing” will become more widely applied in data analyses.

**Key Points**

- A principled not available (NA) deconvolution model: biologically informative NA deconvolution (BIND) uses adaptive learning to classify NAs at the instance level, outperforming global imputation and preserving true biological specificity.
- Enhanced clinical discovery: Across hepatocellular carcinoma tissues and cancer cell line proteomes, BIND identified clinically relevant candidate protein markers such as GPC3 and KPNA2.
- Improved network biology: By incorporating NA information, our missing-value-weighted correlation coefficient (*ρ*_BIND_) substantially enhances protein–protein interaction detection, revealing complexes and pathways that are missed by conventional correlation analyses.
- Broad accessibility through a public web server: The BIND web server enables user-friendly, interactive visualization, and data processing for both bulk and single-cell proteomics datasets, making these tools accessible to researchers without programming expertise.

## Supplementary Material

supplementary_materials_bbag582

## Data Availability

All data and code related to this paper have been uploaded to GitHub repositories (https://github.com/guowh1999/BIND) and uploaded to the online database Zenodo (https://doi.org/10.5281/zenodo.15766996). A user tutorial is provided. The address of the BIND online web server is https://bind.fengslab.com/.

## References

[1] Zhang YY, Fonslow BR, Shan B et al. Protein analysis by shotgun/bottom-up proteomics. *Chem Rev* 2013;113:2343–94. 10.1021/cr3003533 PMC3751594 23438204

[2] Li MB, Smyth GK. Neither random nor censored: estimating intensity-dependent probabilities for missing values in label-free proteomics. *Bioinformatics* 2023;39:btad200. 10.1093/bioinformatics/btad200 PMC10174703 37067487

[3] Folch-Fortuny A, Arteaga F, Ferrer A. PCA model building with missing data: new proposals and a comparative study. *Chemom Intell Lab Syst* 2015;146:77–88. 10.1016/j.chemolab.2015.05.006

[4] Datta S, Bhattacharjee S, Das S. Clustering with missing features: a penalized dissimilarity measure based approach. *Mach Learn* 2018;107:1987–2025. 10.1007/s10994-018-5722-4

[5] Vanderaa C, Gatto L. Revisiting the thorny issue of missing values in single-cell proteomics. *J Proteome Res* 2023;22:2775–84. 10.1021/acs.jproteome.3c00227 37530557

[6] Pedersen AB, Mikkelsen EM, Cronin-Fenton D et al. Missing data and multiple imputation in clinical epidemiological research. *Clin Epidemiol* 2017;9:157–66. 10.2147/CLEP.S129785 PMC5358992 28352203

[7] Lazar C, Gatto L, Ferro M et al. Accounting for the multiple natures of missing values in label-free quantitative proteomics data sets to compare imputation strategies. *J Proteome Res* 2016;15:1116–25. 10.1021/acs.jproteome.5b00981 26906401

[8] Gardner ML, Freitas MA. Multiple imputation approaches applied to the missing value problem in bottom-up proteomics. *Int J Mol Sci* 2021;22. 10.3390/ijms22179650 PMC8431783 34502557

[9] Kong WJ, Hui HWH, Peng H et al. Dealing with missing values in proteomics data. *Proteomics* 2022;22:e2200092. 10.1002/pmic.202200092 36349819

[10] Bramer LM, Irvahn J, Piehowski PD et al. A review of imputation strategies for isobaric labeling-based shotgun proteomics. *J Proteome Res* 2021;20:1–13. 10.1021/acs.jproteome.0c00123 PMC8996546 32929967

[11] Lin MH, Wu PS, Wong TH et al. Benchmarking differential expression, imputation and quantification methods for proteomics data. *Brief Bioinform* 2022;23:bbac138. 10.1093/bib/bbac138 35397162

[12] Egert J, Brombacher E, Warscheid B et al. DIMA: data-driven selection of an imputation algorithm. *J Proteome Res* 2021;20:3489–96. 10.1021/acs.jproteome.1c00119 34062065

[13] Webel H, Niu LL, Nielsen AB et al. Imputation of label-free quantitative mass spectrometry-based proteomics data using self-supervised deep learning. *Nat Commun* 2024;15:5405. 10.1038/s41467-024-48711-5 PMC11208500 38926340

[14] McGurk KA, Dagliati A, Chiasserini D et al. The use of missing values in proteomic data-independent acquisition mass spectrometry to enable disease activity discrimination. *Bioinformatics* 2020;36:2217–23. 10.1093/bioinformatics/btz898 PMC7141869 31790148

[15] Hoshino A, Kim HS, Bojmar L et al. Extracellular vesicle and particle biomarkers define multiple human cancers. *Cell* 2020;182:1044–1061.e18. 10.1016/j.cell.2020.07.009 PMC7522766 32795414

[16] Qiu P . Embracing the dropouts in single-cell RNA-seq analysis. *Nat Commun* 2020;11:1169. 10.1038/s41467-020-14976-9 PMC7054558 32127540

[17] Kryszkiewicz M . A new approach to deriving Jaccard similarity and Jaccard distance properties with and without considering feature weights. In: *16th Asian Conference on Intelligent Information and Database Systems (ACIIDS)*; *Apr 15–18; Amer Univ Ras Al Khaimah, French SIGAPP Chapter, Ras Al Khaimah, U ARAB EMIRATES*, pp. 341–9, 2024.

[18] Konecny J, Trnecka M. Boolean matrix factorization for symmetric binary variables. *Knowl-Based Syst* 2023;279:110944. 10.1016/j.knosys.2023.110944

[19] Feng XK, Chen LX, Wang ZS et al. I-impute: a self-consistent method to impute single cell RNA sequencing data. *BMC Genomics* 2020;21:21. 10.1186/s12864-020-07007-w PMC7677776 33208097

[20] Chen LS, Buja A. Stress functions for nonlinear dimension reduction, proximity analysis, and graph drawing. *J Mach Learn Res* 2013;14:1145–73.

[21] Saleem S, Sajid MS, Varghese RS et al. Phosphopeptidome profiling of human plasma for hepatocellular carcinoma biomarker discovery. *J Proteome Res* 2025;25:1115–25. 10.1021/acs.jproteome.5c01004 PMC12831616 41474787

[22] He L, Sarkar SK. On improving some adaptive BH procedures controlling the FDR under dependence. *Electron J Stat* 2013;7:2683–701. 10.1214/13-EJS855

[23] Raffield LM, Dang H, Pratte KA et al. Comparison of proteomic assessment methods in multiple cohort studies. *Proteomics* 2020;20:e1900278. 10.1002/pmic.201900278 PMC7425176 32386347

[24] Eden SK, Li C, Shepherd BE. Nonparametric estimation of Spearman's rank correlation with bivariate survival data. *Biometrics* 2022;78:421–34. 10.1111/biom.13453 PMC8453584 33704769

[25] Du P, Li FL, Shao JL. Multi-agent reinforcement learning clustering algorithm based on silhouette coefficient. *Neurocomputing* 2024;596:127901. 10.1016/j.neucom.2024.127901

[26] Sundqvist M, Chiquet J, Rigaill G. Adjusting the adjusted Rand index a multinomial story. *Comput Stat* 2023;38:327–47. 10.1007/s00180-022-01230-7

[27] Crosara KTB, Moffa EB, Xiao YZ et al. Merging in-silico and in vitro salivary protein complex partners using the STRING database: a tutorial. *J Proteome* 2018;171:87–94. 10.1016/j.jprot.2017.08.002 28782718

[28] Goncalves E, Poulos RC, Cai Z et al. Pan-cancer proteomic map of 949 human cell lines. *Cancer Cell* 2022;40:835–849.e8. 10.1016/j.ccell.2022.06.010 PMC9387775 35839778

[29] Gholami AM, Hahne H, Wu ZX et al. Global proteome analysis of the NCI-60 cell line panel. *Cell Rep* 2013;4:609–20. 10.1016/j.celrep.2013.07.018 23933261

[30] Frejno M, Meng C, Ruprecht B et al. Proteome activity landscapes of tumor cell lines determine drug responses. *Nat Commun* 2020;11:3639. 10.1038/s41467-020-17336-9 PMC7371697 32686665

[31] Jiang Y, Sun AH, Zhao Y et al. Proteomics identifies new therapeutic targets of early-stage hepatocellular carcinoma. *Nature* 2019;567:257–61. 10.1038/s41586-019-0987-8 30814741

[32] Sanchez-Avila X, Truong T, Xie XF et al. Easy and accessible workflow for label-free single-cell proteomics. *J Am Soc Mass Spectrom* 2023;34:2374–80. 10.1021/jasms.3c00240 PMC11002963 37594399

[33] Yuan YD, Chao M, Lo YC. Automatic skin lesion segmentation using deep fully convolutional networks with Jaccard distance. *IEEE Trans Med Imaging* 2017;36:1876–86. 10.1109/TMI.2017.2695227 28436853

[34] Wryk G, Gawor A, Bulska E. Comprehensive evaluation of advanced imputation methods for proteomic data acquired via the label-free approach. *Int J Mol Sci* 2024;25:25. 10.3390/ijms252413491 PMC11728106 39769253

[35] Mou XY, Du HY, Qiao GH et al. Evaluation of imputation and imputation-free strategies for differential abundance analysis in metaproteomics data. *Brief Bioinform* 2025;26:26. 10.1093/bib/bbaf141 PMC12009712 40254829

[36] Zhang SC . Nearest neighbor selection for iteratively kNN imputation. *J Syst Softw* 2012;85:2541–52. 10.1016/j.jss.2012.05.073

[37] Güngör E, Özmen A. Distance and density based clustering algorithm using Gaussian kernel. *Expert Syst Appl* 2017;69:10–20. 10.1016/j.eswa.2016.10.022

[38] Zheng XF, Liu X, Lei YN et al. Glypican-3: a novel and promising target for the treatment of hepatocellular carcinoma. *Front Oncol* 2022;12. 10.3389/fonc.2022.824208 PMC8889910 35251989

[39] Shi DH, Shi YP, Kaseb AO et al. Chimeric antigen receptor-glypican-3 T-cell therapy for advanced hepatocellular carcinoma: results of phase I trials. *Clin Cancer Res* 2020;26:3979–89. 10.1158/1078-0432.CCR-19-3259 32371538

[40] Liu MY, Dongre A. Proper imputation of missing values in proteomics datasets for differential expression analysis. *Brief Bioinform* 2021;22:bbaa112. 10.1093/bib/bbaa112 32520347

[41] Lin WQ, Ji JD, Su KJ et al. Deng HW: omicsMIC: a comprehensive benchmarking platform for robust comparison of imputation methods in mass spectrometry-based omics data. *Nar Genom Bioinform* 2024;6:lqae071. 10.1093/nargab/lqae071 PMC11177553 38881578

[42] Lalande F, Doya K. Numerical data imputation: Choose kNN over deep learning. In: *15th International Conference on Similarity Search and Applications (SISAP)*; *Oct 05–07*, pp. 3–10. Bologna, ITALY: Univ Bologna, 2022.

[43] Kong WJ, Wong BJH, Gao HH et al. PROTREC: a probability-based approach for recovering missing proteins based on biological networks. *J Proteome* 2022;250:104392. 10.1016/j.jprot.2021.104392 34626823

[44] Gromley A, Yeaman C, Rosa J et al. Centriolin anchoring of exocyst and SNARE complexes at the midbody is required for secretory-vesicle-mediated abscission. *Cell* 2005;123:75–87. 10.1016/j.cell.2005.07.027 16213214

[45] Hamasaki M, Furuta N, Matsuda A et al. Autophagosomes form at ER-mitochondria contact sites. *Nature* 2013;495:389–93. 10.1038/nature11910 23455425

[46] Zhang YT, Qin PF, Tian LL et al. The role of mediator complex subunit 19 in human diseases. *Exp Biol Med* 2021;246:1681–7. 10.1177/15353702211011701 PMC8719036 34038190

[47] Sun LM, Liu YF, Guo XY et al. Acetylation-dependent regulation of core spliceosome modulates hepatocellular carcinoma cassette exons and sensitivity to PARP inhibitors. *Nat Commun* 2024;15:5209. 10.1038/s41467-024-49573-7 PMC11189467 38890388

[48] Azzariti A, Mancarella S, Porcelli L et al. Hepatic stellate cells induce hepatocellular carcinoma cell resistance to sorafenib through the laminin-332/α3 integrin axis recovery of focal adhesion kinase ubiquitination. *Hepatology* 2016;64:2103–17. 10.1002/hep.28835 27639064

[49] Tümen D, Heumann P, Gülow K et al. Pathogenesis and current treatment strategies of hepatocellular carcinoma. *Biomedicines* 2022;10:3202. 10.3390/biomedicines10123202 PMC9775527 36551958

