# Supplementary material for: Biologically informative not available deconvolution excavates hidden features in proteomics datasets
Source: Brief Bioinform. 2026 Oct 26;27(5):bbag582. doi: 10.1093/bib/bbag582 (PMC13704793; doi:10.1093/bib/bbag582)

Supplementary Figures of

Biologically Informative NA Deconvolution (BIND) excavates hidden features in proteomics datasets

Fig S1. NA heatmap of human cell line dataset (A)NCI60 and (B)CRC65. Blue cells represent NAs, and Red cells represent non-NAs(expression value)

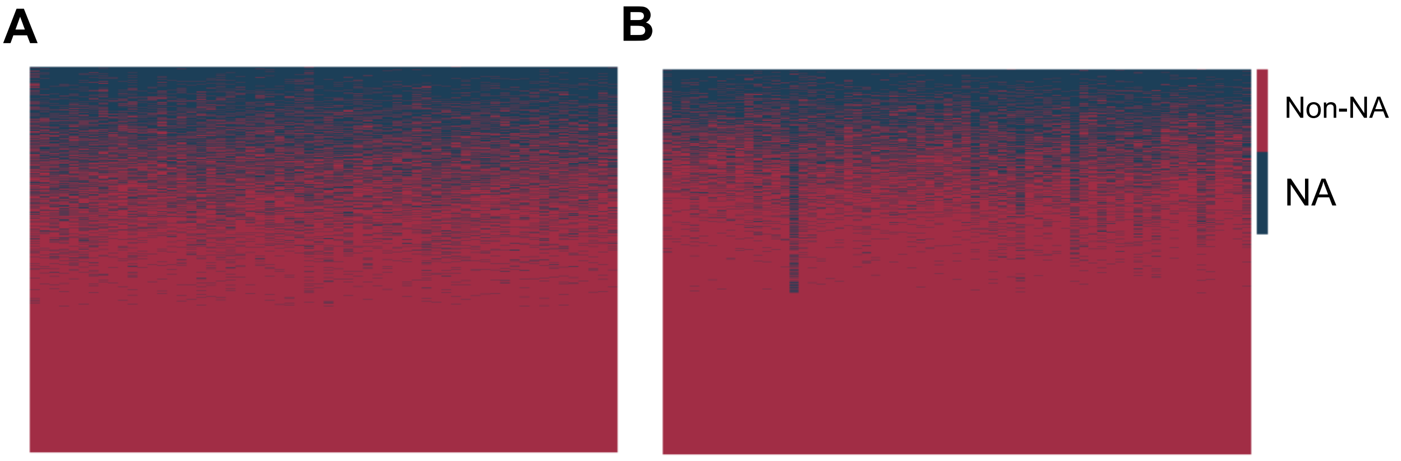

Fig S2. NA proportion histogram of human cell line dataset (A)NCI60 and (B)CRC65. The x-axis represents the NA proportion, and the y-axis represents the protein percentage corresponding to that NA proportion.

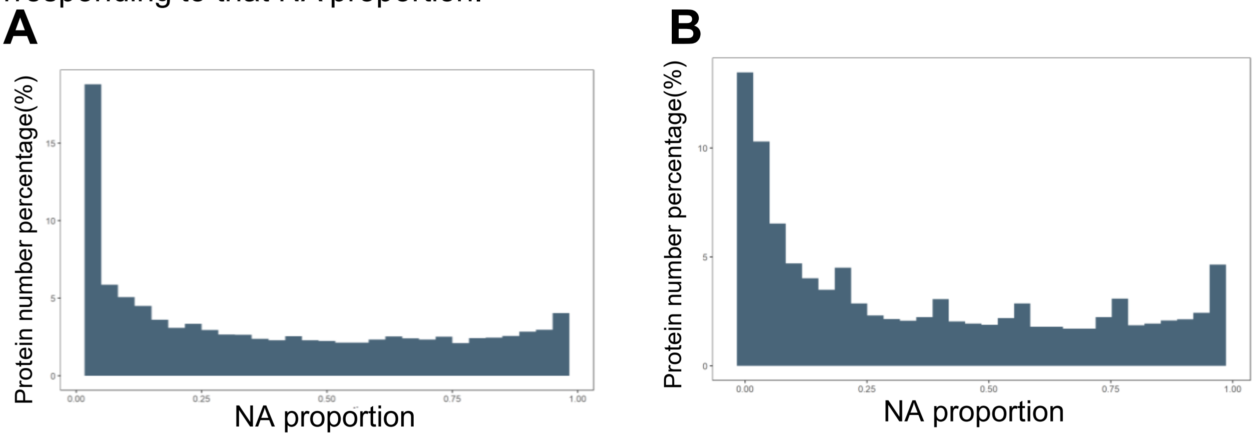

Fig S3. Low NA proportion proteins have high expression values in individual cell lines. (A) HeLa; (B) HCT-116; (C) A549; (D) NALM-6. The x-axis represents the proteins, sorted in ascending order in the sample. The y-axis represents the expression level (mass spectrometry intensity) of that protein.

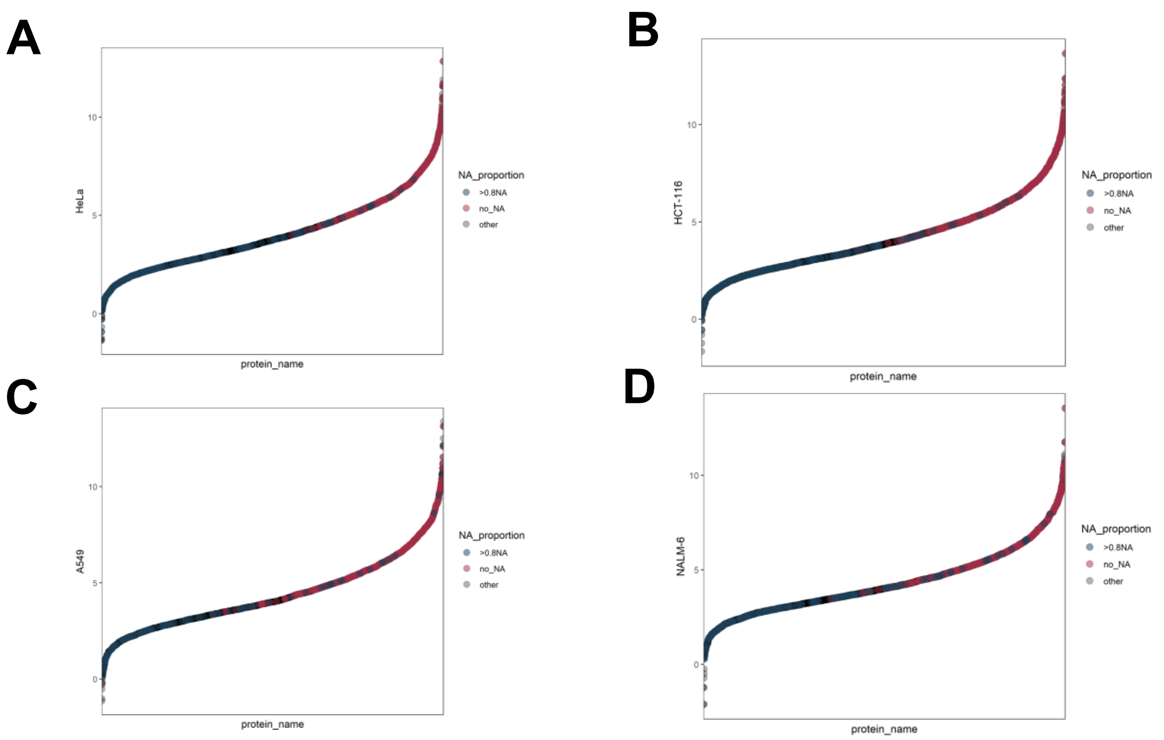

Fig S4. Sample numbers of each tissue in CCLE949 dataset. The x-axis is sample number.

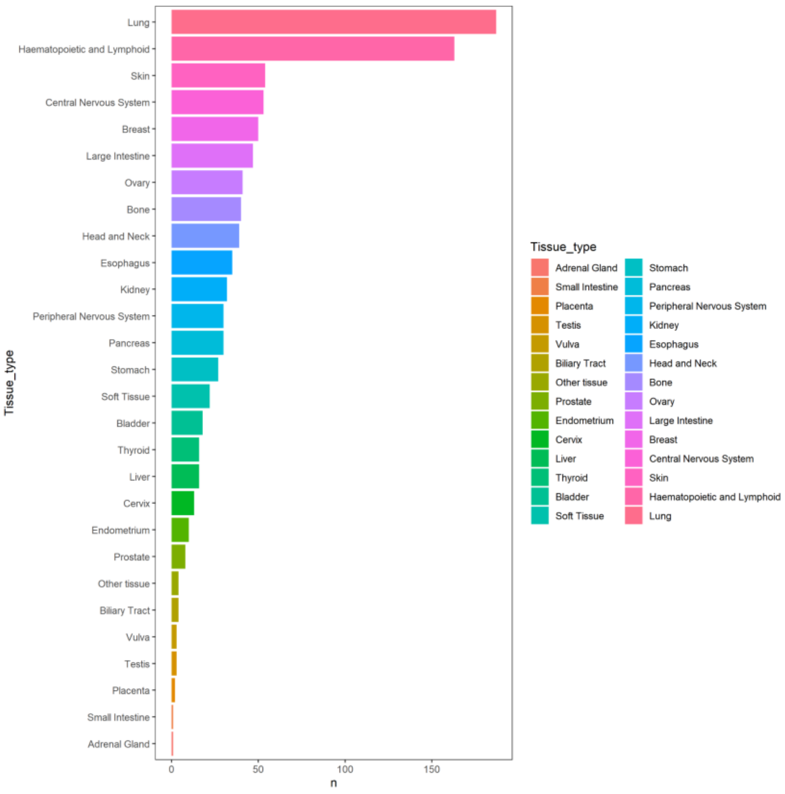

Fig S5. Clustering of CCLE949 samples by the Euclidean distance with the numeric data matrix after removing all NAs.

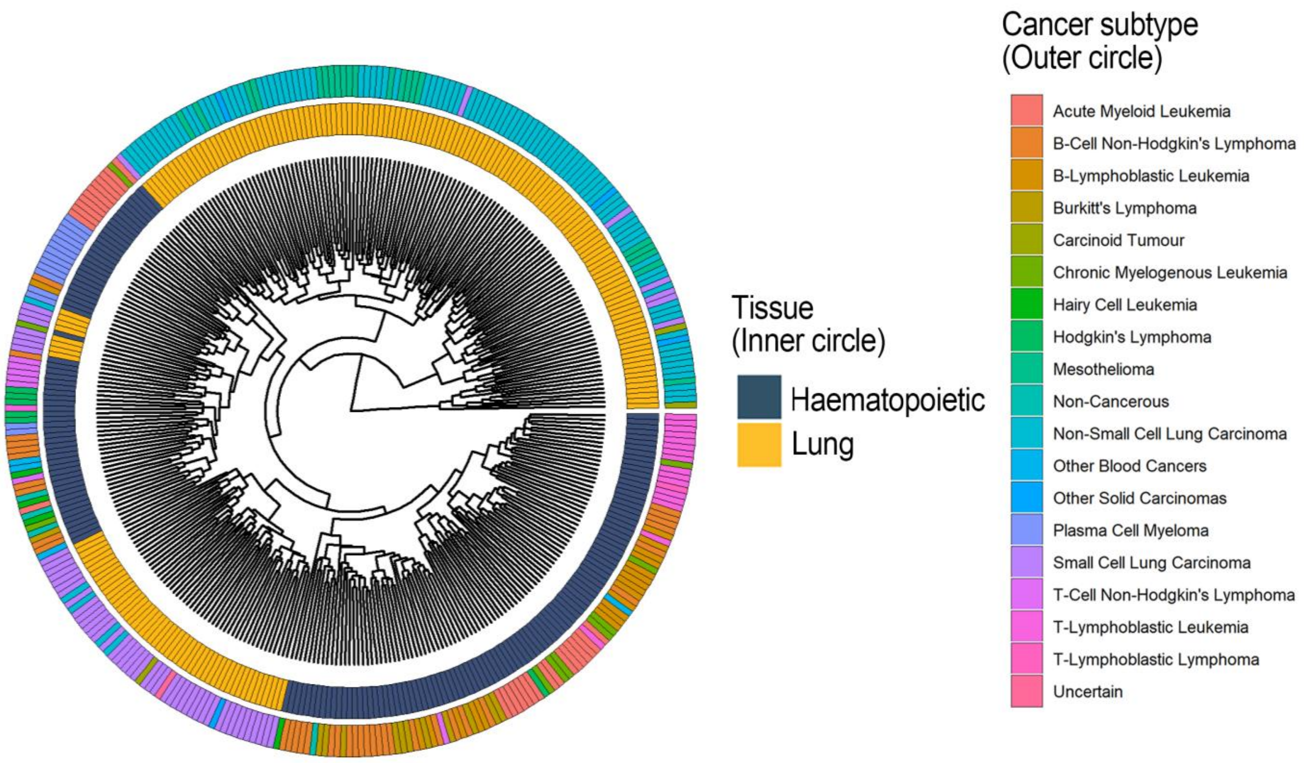

Fig S6. GO pathway enrichment of proteins **absent** in hematopoietic tumors (present in solid tumors).

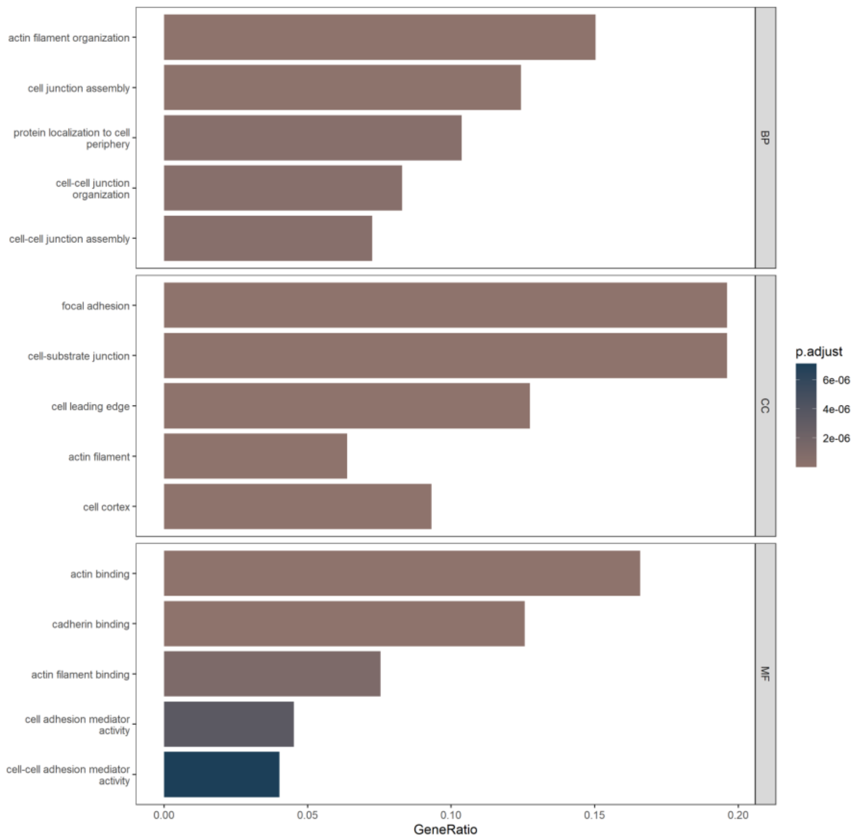

Fig S7. GO pathway enrichment of proteins **present** in hematopoietic tumors (absent in solid tumors).

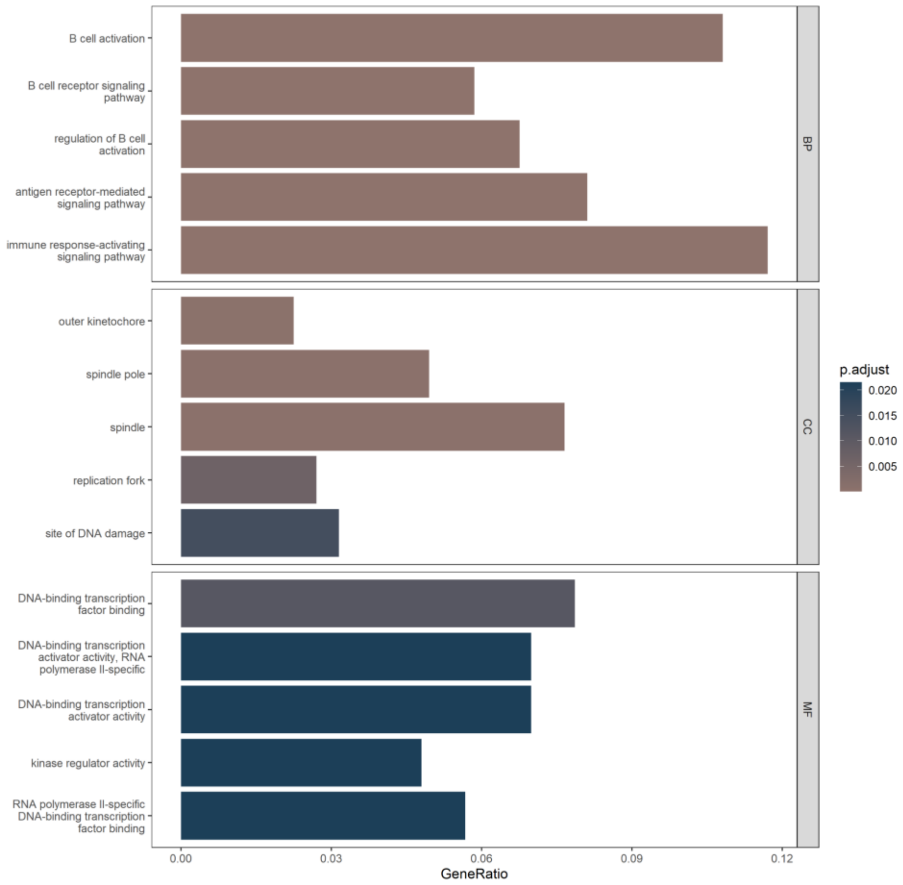

Fig S8. NA classification ROC curve of different k in the simulation dataset. The x-axis represents the True Positive Rate, and the y-axis represents the False Positive Rate.

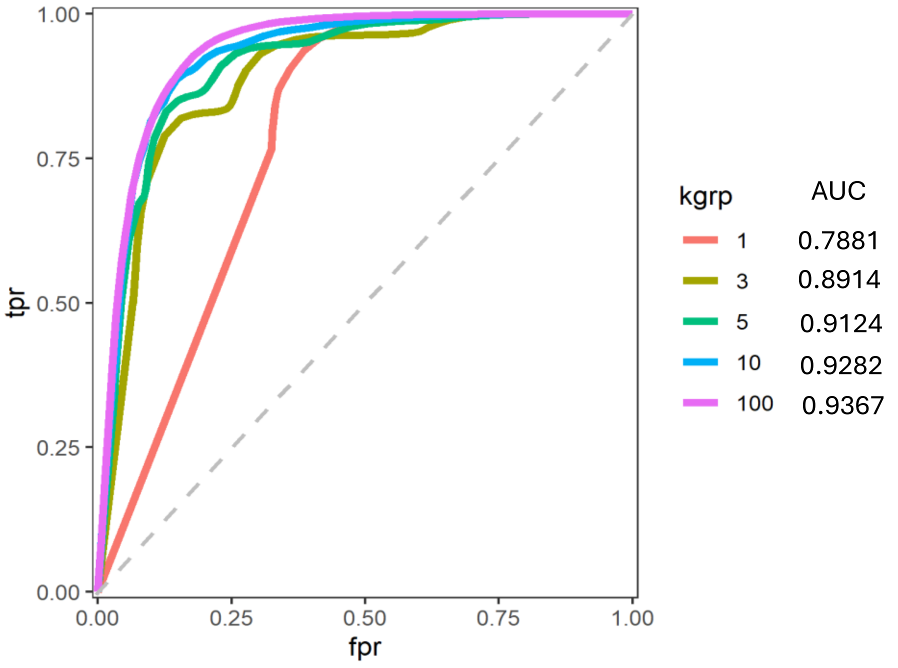

Fig S9. (A) MDS dimension reduction results and (B) silhouette coefficient and ARI for KNN-imputed data at k=3, k=10 and k=50

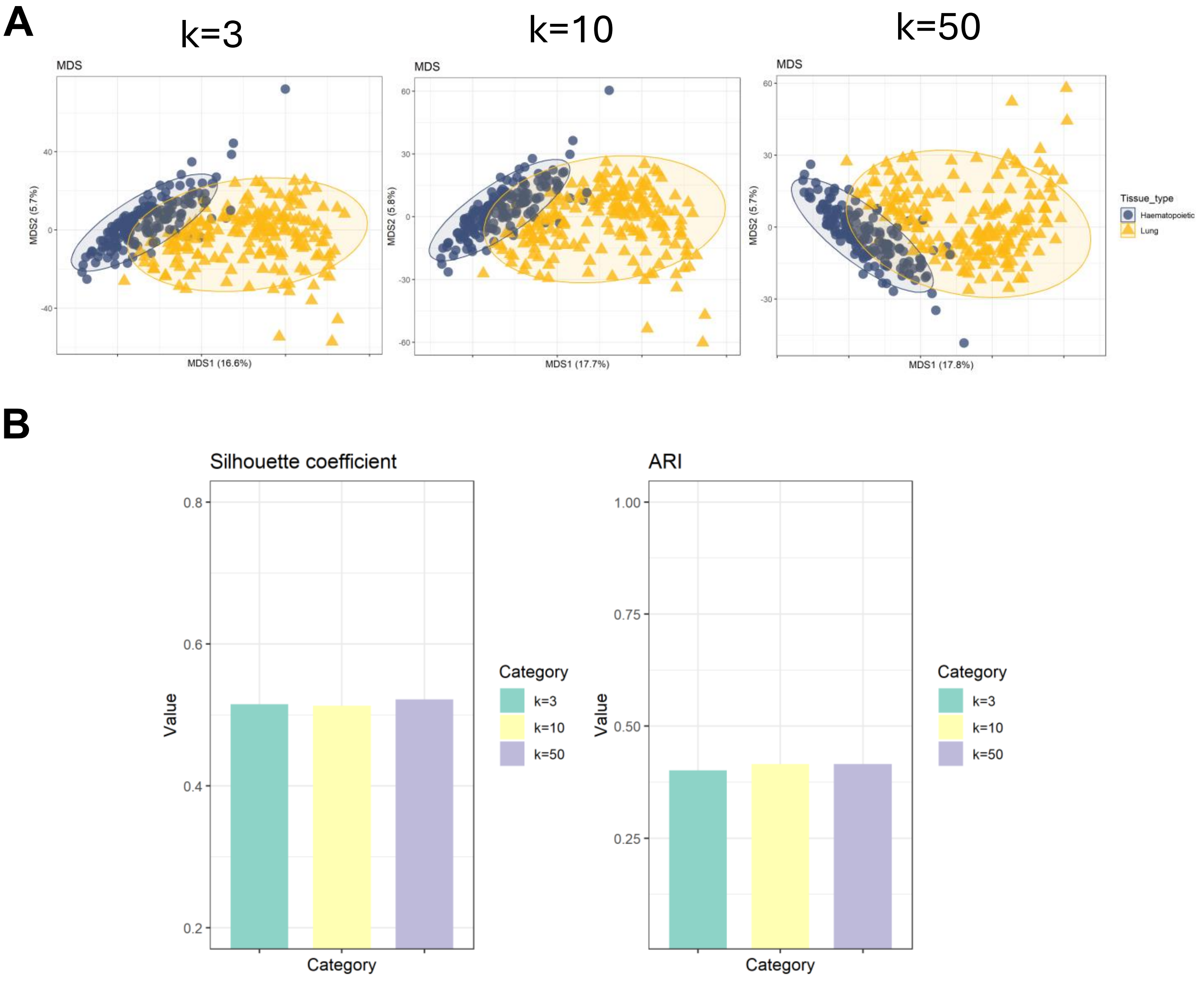

Fig S10. MDS results for various imputation methods applied to data on small cell lung cancer and non-small cell lung cancer.

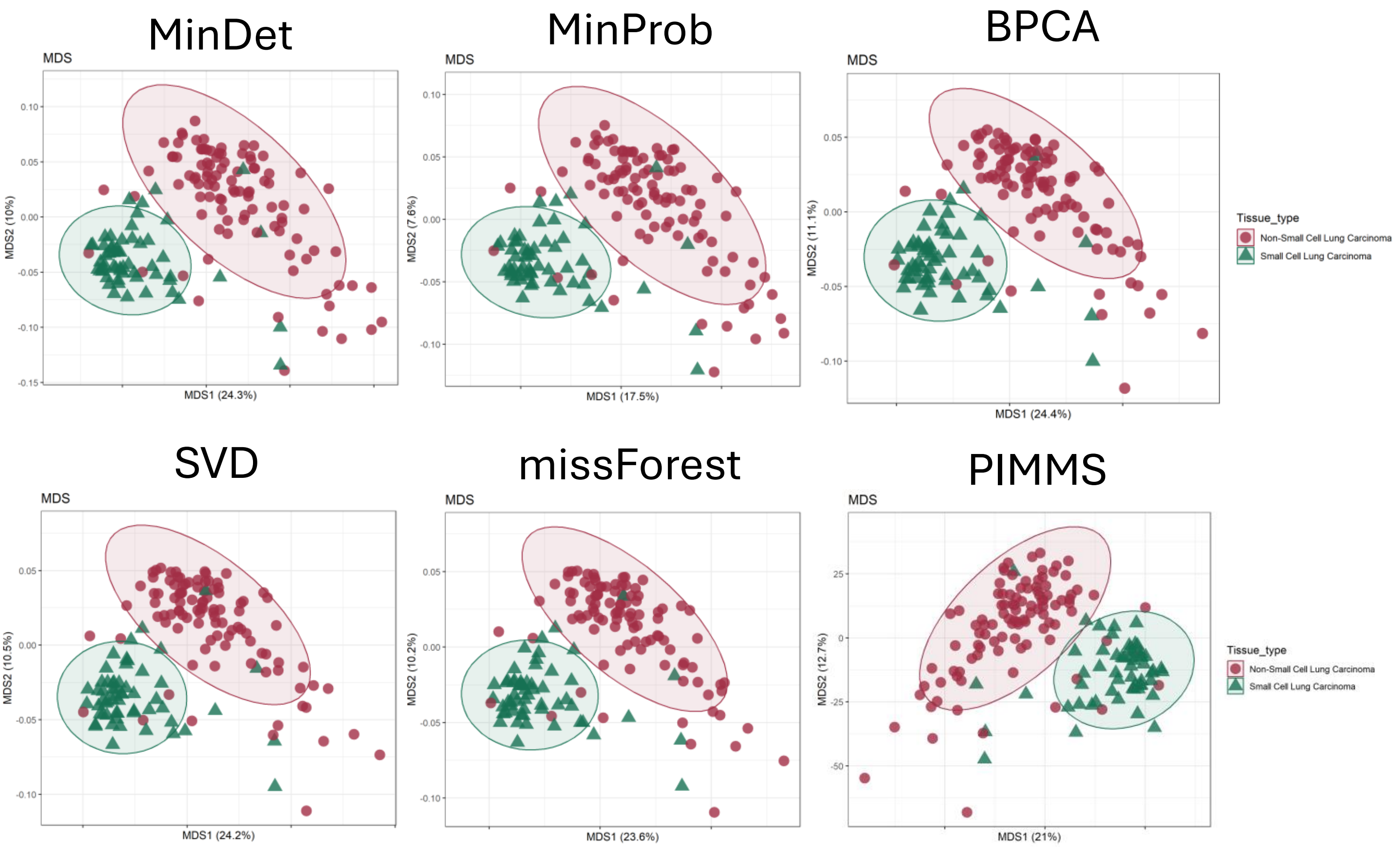

Fig S11. By improving on Spearman ρ, ρ_{BIND} rises (correlates more strongly) over Spearman ρ in a higher proportion of protein pairs with PPIs than without PPIs. The x-axis indicates protein pairs with or without PPI. The y-axis shows the percentage increase or decrease in ρ_{BIND} compared to Spearman ρ.

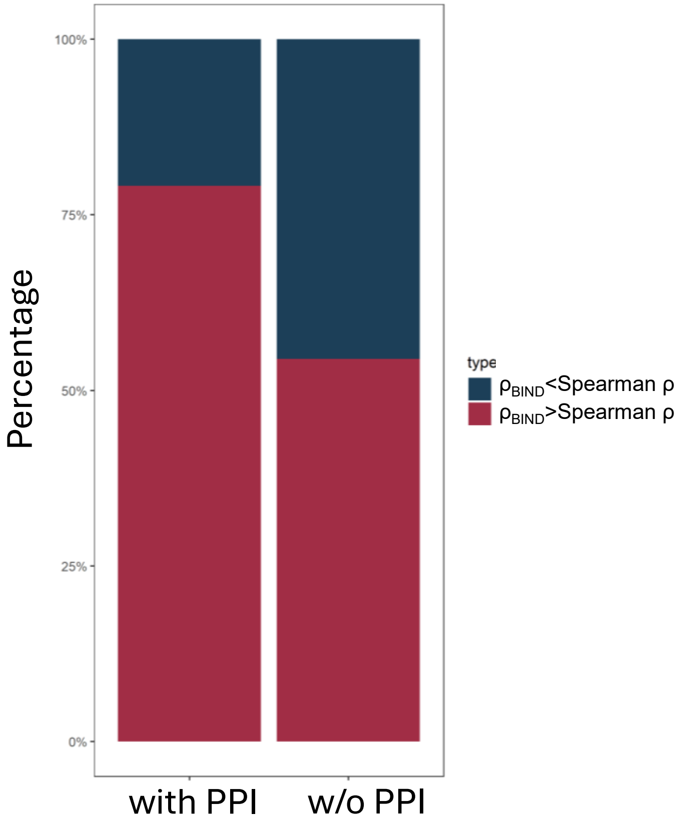

Fig S12. ρ_{BIND} found more PPIs in (A)EXOC complex, (B)MED complex and (C)STX complex. The upper-left portion of the matrix represents ρ_{BIND}, and the lower-right portion represents the original Spearman's ρ.

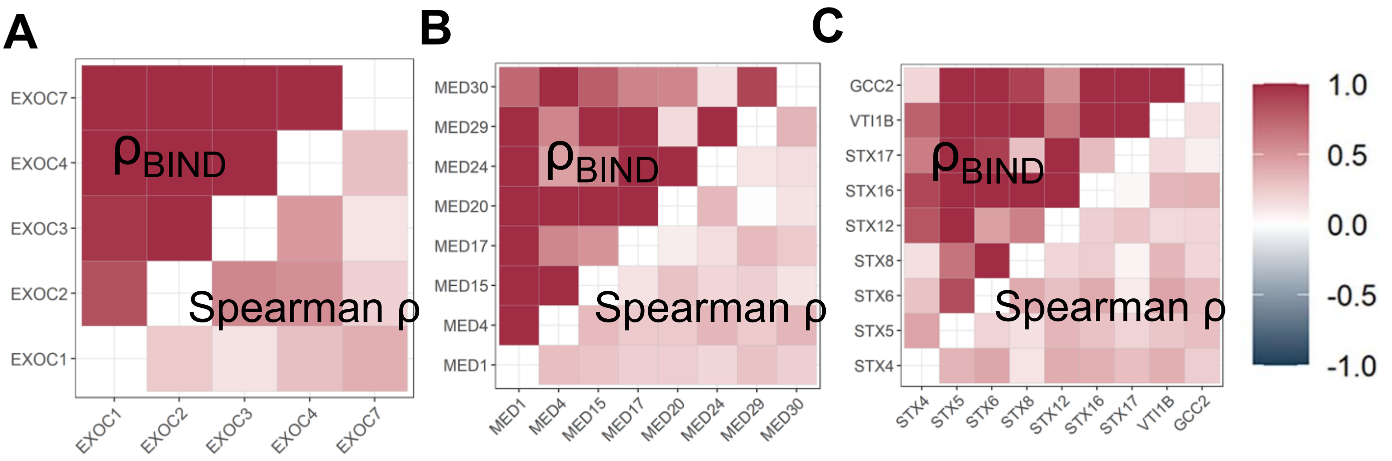

Fig S13. KEGG pathway enrichment of HCC markers.

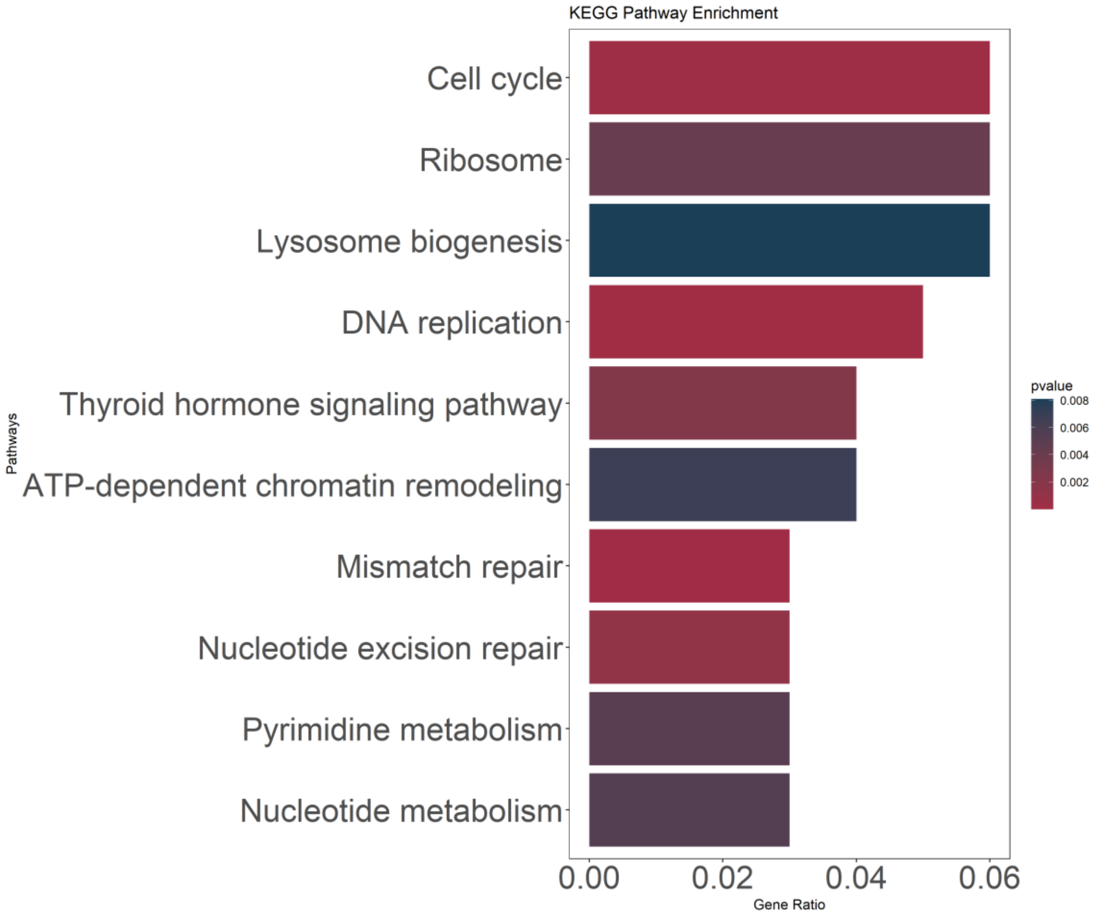

Fig S14. GO enrichment analysis of jurkat cell line markers found by BIND web server.

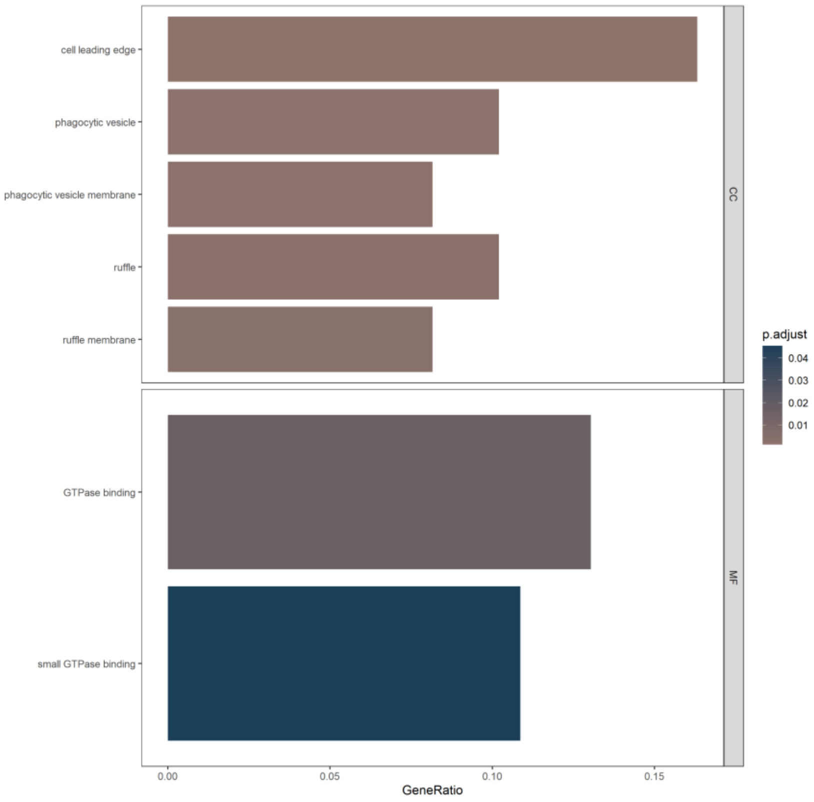

Supplement: Supplementary file 1 [file supplementary_materials_bbag582.zip › SFigures_rev.pdf]
