# Supplementary material for: Biologically informative not available deconvolution excavates hidden features in proteomics datasets
Source: Brief Bioinform. 2026 Oct 26;27(5):bbag582. doi: 10.1093/bib/bbag582 (PMC13704793; doi:10.1093/bib/bbag582)
Supplement: Supplementary file 1 [file supplementary_materials_bbag582.zip › SupplementaryMethod_final.docx]

Supplementary methods of

**Biologically Informative NA Deconvolution (BIND) excavates hidden features in proteomics datasets**

**Data collection and acquisition**

Five large-scale proteomics datasets were used in this study. Human cancer cell line datasets were used for method establishment and evaluation [1-3]. A human liver cancer and paired normal tissue dataset (HCC) was used to identify the disease marker [4]. In addition, we use a single-cell proteomic (SCP) dataset to evaluate our method at the single-cell level [5].

*CCLE949.* Quantitative DIA analysis was performed on 949 cell lines from 40 cancer types in 28 tissues. The resulting dataset was derived from 6,864 DIA-MS runs acquired over 10,000 MS h, including HEK293T for all data acquisition cycles and quality control instrumentation. SWATH-MS measurements were performed on a 6600 TripleTOF system. A total of 6,692 human proteins were quantified.

*NCI60 and CRC65.* NCI-60 consists of 59 individual cancer cell lines from 9 different tissues. These cells were lysed and digested with trypsin into a nanoLC-Orbitrap Velos mass spectrometer for analysis. Data-dependent acquisition (DDA) was used in positive ion mode. The CRC65 data contains proteomics datasets of 65 colorectal cancer cell lines with the same data acquisition process as NCI60.

*Hepatocellular carcinoma (HCC).* The HCC dataset originates from surgically resected hepatocellular carcinoma tissue and paired non-tumor liver tissue. Minced liver tissue and liver cells were lysed, and protein concentrations were measured prior to trypsin digestion. Liquid chromatography separation was performed using the Easy nLC1000 system, with mass spectrometry scanning conducted on the Orbitrap Fusion.

*Single-cell proteomics (SCP).* Single-cell proteomics can provide proteomics data on a larger scale and with higher resolution. We used a Hela and Jurkat cell line SCP dataset. Hela and Jurkat cell cultures were performed in vitro, and single-cell fractionators were used to generate single cells. Cell lysis, protein denaturation, and digestion were performed using a mixture of trypsin and LysC digests. NanoLC separations were performed using an UltiMate 3000 RSLCnano system with a SPE column. MS analysis was performed using an Orbitrap Exploris 480 equipped with a Nanospray Flex Ion Source.

**Experiment details**

*NA classification method evaluation.* We evaluated NA classification methods using a proteomics dataset from multiple human cancer cell lines. This dataset contains 949 cell line samples. First, simulated dataset was generated based on the aforementioned simulation approach. When applying BIND, we adjusted the neighbor parameter $k$ and threshold $\gamma$ while observing changes in classification accuracy. After fixing $k$, we dynamically adjusted $\gamma$ to construct ROC curves for evaluating classification performance. The ratios of “biological” NA to “technical” NA in the simulation data were set to 3:1, 2:1, 1:1, 1:2, and 1:3 to evaluate their influence on classification performance. In addition, we compared the accuracy of NA classification across three NA proportion groups—low (0–0.4), middle (0.4–0.6), and high (0.6–1)—to assess the impact of NA proportion on the results.

To assess the contributions of neighborhood topology and local expression information to BIND classification, we performed a sequential shuffling ablation. The original, unshuffled data were used as the baseline. We first randomized the neighborhood topology used for NA classification while retaining the expression values. Starting from this neighborhood-shuffled condition, we then additionally shuffled the expression values within the randomized neighborhoods. Thus, the two perturbations were applied sequentially and cumulatively rather than independently, and the final condition represents disruption of both neighborhood topology and within-neighborhood expression information.

*Sample dimensionality reduction and clustering.* After applying BIND to the entire dataset, our first biological task is sample dimensionality reduction and clustering. We employed MDS dimension reduction based on Jaccard distance to reduce the dimensionality of the binary dataset. For comparison, we obtained the data matrix after removing all rows and columns that contain NA, as well as the data matrix by performing kNN data imputation on the original dataset [6]. We then reduced their dimensionality using MDS with Euclidean distance. We also evaluated the results of calculating the Jaccard distance and performing clustering on the raw data after binarization to verify that BIND’s performance does not depend entirely on the original missing value pattern.

For the reduced-dimensional data, we employed k-means clustering to group the data and examined the discrepancies between the clustering results and the actual categories. We performed 50 random initializations to achieve optimal outcomes. The results were evaluated using the Silhouette Coefficient [7] and the Adjusted Rand Index (ARI) [8].

We also masked the missing value classification results based on the NA proportion of the proteins to assess which range of NA proportions has a greater impact on the dimension reduction effect. Specifically, in the binary matrix of BIND results, proteins with an NA proportion < n% were uniformly set to 1, thereby eliminating differences among samples. n was initially set to 10 and increased to 90.

*Benchmark with imputation methods.* To further illustrate BIND's capabilities, we compared BIND with various imputation methods. We referenced the methods described in NAguideR, a proteomics data imputation tool, and used representative imputation methods as benchmarks. The methods we used include the univariate methods MinDet and MinProb, the global methods SVD and BPCA, and the local methods kNN and missForest. In addition, PIMMS, a recently published proteomics imputation method that utilizes advanced deep learning models, has been included in our benchmark [9]. For imputation methods that require parameter selection, we tested different parameters for each method and selected the parameters that yielded the best downstream clustering results. In particular, for KNN imputation, we demonstrated the clustering performance for various values of the parameter k in our paper. The detailed final parameter settings for each method (including BIND) are as follows, and unlisted parameters retain their default settings:

**Table S1 Final parameter settings and implementation of BIND and benchmark methods**

| Method | Final parameter setting | Software | Package | Function |
| --- | --- | --- | --- | --- |
| MinDet | q=0.01 | *R 4.5.3* | *imputeLCMD 2.1* | *impute.MinDet* |
| MinProb | q=0.01, tune.sigma = 1 | *R 4.5.3* | *imputeLCMD 2.1* | *impute.MinProb* |
| SVD | K=5 | *R 4.5.3* | *imputeLCMD 2.1* | *impute.wrapper.SVD* |
| BPCA | nPcs=8, scale=TRUE,  method=”bpca”,  center=TRUE | *R 4.5.3* | *pcaMethods 2.2.0* | *pca* |
| kNN | k=10 | *R 4.5.3* | *Impute 1.84.0* | *impute.knn* |
| missForest | ntree=100, maxiter=10 | *R 4.5.3* | *missForest 1.6.1* | *missForest* |
| PIMMS | latent_dim=50, hidden_layers=[512],  batch_size=10,  model=”VAE” | *Python 3.10.0* | *torch 2.11.0*  *pimms-learn 0.5.2* | *AETransformer* |
| BIND | k=10, γ =3 | *R 4.5.3* | / | / |

For BIND, the neighborhood size *k* and classification threshold *γ* were selected by jointly considering NA-classification performance and computational burden. In the method evaluation in this paper, the parameter ultimately used was the near-optimal option *k*=10 and *γ*=3. For kNN imputation, the number of nearest neighbors was set to *k_kNN-imp_*=10 for the reported benchmark analyses. Sensitivity analyses over alternative *k_kNN-imp_* settings are provided in the corresponding supplementary figures.

*Marker identification.* The aforementioned method was used to identify group-specific biomarkers. In the cell line dataset, a |log2FC| $\geq$ 2 and adjusted p-value $\leq$ 10^-35^ were established as the thresholds for biomarker identification. In addition, we directly compared the markers identified by BIND and those identified by kNN imputation combined with differential analysis. In the kNN imputation data, log2FC employed a threshold of 1, and the adjusted p-value used a threshold of 0.05. We also compared the expression of BIND-identified markers in cell lines in the Human Protein Atlas database (<https://www.proteinatlas.org/>). The data we used is from the “Cell Line Cancer Group” dataset, which contains RNA expression data for genes in tumor cell lines from different tissues. For the HCC data, these thresholds were set to |log2FC| $\geq$1.5 and adjusted p-value $\leq$ 10^-10^, respectively. The top 100 eligible markers in each group were chosen to generate a clustering heatmap.

*Protein-protein interaction analysis.* The default value for rewards and penalties for the NA pattern was set to 0.01. We extracted human protein pairs with physical interactions and an interaction strength greater than 0.9 from the STRING database as a reference [10]. We examined whether the $\rho_{BIND}$ was higher for protein pairs with existing PPIs compared to those without PPIs. Using a threshold of 0.5, we investigated whether $\rho_{BIND}$ could identify more PPIs within human protein complexes. We also acquired the top 20 protein complexes by number from the CORUM human protein complex database (https://mips.helmholtz-muenchen.de/corum/). We analyzed the proportion of protein interactions that BIND and kNN imputation could identify in each complex. In comparing PPIs between cancer and normal tissues in HCC data, we identified characteristic PPIs for hepatocellular carcinoma with a score greater than 0.7 in cancer tissues and a difference greater than 0.5 between cancer and normal tissues.

[1] Goncalves E, Poulos RC, Cai Z, Barthorpe S, Manda SS, Lucas N, Beck A, Bucio-Noble D, Dausmann M, Hall C, et al: **Pan-cancer proteomic map of 949 human cell lines.** *Cancer Cell* 2022, **40:**835-+.

[2] Gholami AM, Hahne H, Wu ZX, Auer FJ, Meng C, Wilhelm M, Kuster B: **Global Proteome Analysis of the NCI-60 Cell Line Panel.** *Cell Reports* 2013, **4:**609-620.

[3] Frejno M, Meng C, Ruprecht B, Oellerich T, Scheich S, Kleigrewe K, Drecoll E, Samaras P, Hogrebe A, Helm D, et al: **Proteome activity landscapes of tumor cell lines determine drug responses.** *Nature Communications* 2020, **11**.

[4] Jiang Y, Sun AH, Zhao Y, Ying WT, Sun HC, Yang XR, Xing BC, Sun W, Ren LL, Hu B, et al: **Proteomics identifies new therapeutic targets of early-stage hepatocellular carcinoma.** *Nature* 2019, **567:**257-+.

[5] Sanchez-Avila X, Truong T, Xie XF, Webber KGI, Johnston SM, Lin HJL, Axtell NB, Puig-Sanvicens V, Kelly RT: **Easy and Accessible Workflow for Label-Free Single-Cell Proteomics.** *Journal of the American Society for Mass Spectrometry* 2023, **34:**2374-2380.

[6] Gardner ML, Freitas MA: **Multiple Imputation Approaches Applied to the Missing Value Problem in Bottom-Up Proteomics.** *International Journal of Molecular Sciences* 2021, **22**.

[7] Du P, Li FL, Shao JL: **Multi-agent reinforcement learning clustering algorithm based on silhouette coefficient.** *Neurocomputing* 2024, **596**.

[8] Sundqvist M, Chiquet J, Rigaill G: **Adjusting the adjusted Rand Index A multinomial story.** *Computational Statistics* 2023, **38:**327-347.

[9] Webel H, Niu LL, Nielsen AB, Locard-Paulet M, Mann M, Jensen LJ, Rasmussen S: **Imputation of label-free quantitative mass spectrometry-based proteomics data using self-supervised deep learning.** *Nature Communications* 2024, **15**.

[10] Crosara KTB, Moffa EB, Xiao YZ, Siqueira WL: **Merging in-silico and in vitro salivary protein complex partners using the STRING database: A tutorial.** *Journal of Proteomics* 2018, **171:**87-94.
